# Assessing Uncertainties of Theoretical Atomic Transition Probabilities with Monte Carlo Random Trials

**DOI:** 10.3390/atoms2020086

**Published:** 2014-04-14

**Authors:** Alexander Kramida

**Affiliations:** National Institute of Standards and Technology, Gaithersburg, MD 20899, USA; Tel.: +1-301-975-8074; Fax: +1-301-975-5560

**Keywords:** atomic spectra, transition probabilities, evaluation of uncertainties

## Abstract

This paper suggests a method of evaluation of uncertainties in calculated transition probabilities by randomly varying parameters of an atomic code and comparing the results. A control code has been written to randomly vary the input parameters with a normal statistical distribution around initial values with a certain standard deviation. For this particular implementation, Cowan’s suite of atomic codes (R.D. Cowan, *The Theory of Atomic Structure and Spectra*, Berkeley, CA: University of California Press, 1981) was used to calculate radiative rates of magnetic-dipole and electric-quadrupole transitions within the ground configuration of titanium-like iron, Fe V. The Slater parameters used in the calculations were adjusted to fit experimental energy levels with Cowan’s least-squares fitting program, RCE. The standard deviations of the fitted parameters were used as input of the control code providing the distribution widths of random trials for these parameters. Propagation of errors through the matrix diagonalization and summation of basis state expansions leads to significant variations in the resulting transition rates. These variations vastly differ in their magnitude for different transitions, depending on their sensitivity to errors in parameters. With this method, the rate uncertainty can be individually assessed for each calculated transition.

## 1. Introduction

With the rapid improvement in computer power and quality of atomic structure codes, calculations of atomic structure and transition properties now become widely used in large-scale simulations of physical conditions in complex plasma environments, such as found in fusion devices. Such simulations have wide range of applications, from plasma diagnostics to prediction of technological characteristics of industrial devices. To assess the accuracy of these simulations, it is important to have well-defined uncertainties for all calculated atomic parameters. Thus, critical evaluation of atomic data, implying estimation of uncertainties, has recently become one of the top priorities in fusion research [1].

Currently existing methods of evaluation of uncertainties of calculated transition probabilities were summarized by Wiese [2] and Kramida [3]. These methods heavily rely on comparisons between different calculations and between calculations and experiments.

In this paper, I suggest a new method of evaluation of uncertainties. It also relies on comparisons; however, these comparisons do not use any external data, but only the data produced by the same computational procedure. The base for comparisons is built from data generated with varied parameters of the atomic code. Application of this method is illustrated for the case of magnetic-dipole (M1) and electric-quadrupole (E2) transitions within the ground configuration of titanium-like iron (Fe V). The calculations were made with the suite of atomic structure codes by Cowan [4]. Uncertainties evaluated with this method are compared with recent critical compilation [5], where radiative rates of these transitions were also calculated with the same Cowan codes, and their uncertainties were evaluated by comparison with calculations of Nahar *et al.* [6].

## 2. Method Description

Calculation of transition probabilities involves several stages. These stages differ in different methods. In the non-relativistic or quasi-relativistic Hartree–Fock method, at first, the radial parts of wavefunctions are computed for each configuration in the single-configuration approximation. Then the Slater parameters and configuration–interaction (CI) parameters, as well as multipole transition integrals, are calculated from these radial functions. The parameters mentioned above are called hereafter the *input parameters*. The Hamiltonian matrix is built from these input parameters and diagonalized. The resulting eigenvalues and eigenvectors represent the initial calculated energy levels. Then these parameters are varied in the least-squares fitting (LSF) to find the set of parameters that best fits the experimental energy levels. Then these LSF parameters are used as input for the matrix diagonalization procedure, producing an improved set of eigenvalues and eigenvectors. These are used to compute the multipole transition probabilities. In most cases, because of the limited accuracy of the approximate atomic model, the LSF procedure does not exactly reproduce the experimental energy levels. Since the derivatives of the eigenvalues over the Slater and CI parameters can readily be calculated, the uncertainties (standard deviations, hereafter denoted as *σ*) of the LSF parameters can be computed from the differences between the fitted and experimental energies.

The idea of the method is to vary the LSF parameters in a random fashion (using a normal statistical distribution centered at the LSF values with a width equal to the *σ* of the LSF) and to evaluate the standard (root-mean-square) deviation of the line strengths calculated with these varied parameters.

It must be noted that this initial assumption of normal statistical distributions of all parameters is arbitrary to a large degree. One could assume, for example, that these distributions are uniform, meaning that all values of parameters within certain limits are equally probable. However, a normal statistical distribution seems to better reflect the common observation that the results of the LSF provide the best (most probable) values for the parameters insofar as the eigenvalues obtained with the fitted parameters exhibit symmetrical distributions around experimental values with the smallest standard deviations. The standard distribution has well-known statistical properties such as its symmetry around the central value (mean), its width at half-maximum (equal to 1.35 times the standard deviation around the mean) and the probabilities of occurrence of values deviating by more than one, two, or three standard deviations (32%, 5%, and 0.3%, respectively). Although other forms of distributions are possible, the normal distribution seems to be a good test case.

Cowan’s codes [4] already have all necessary routines such as the LSF. Thus, this suite of codes was chosen as a test platform for the suggested method.

A few preliminary remarks should be made. The first one concerns the statistical distributions of the calculated quantities. In several recent papers, including [3], I made a statement that the logarithm of the calculated line strength, log(*S*), has much better statistical properties than the line strength itself, in the sense that the statistical distribution of log(S/S*), where S* is a true value, is much closer to a normal distribution than S/S*. It turns out that this statement is generally incorrect. As discussed in Section 3, for the considered M1 and E2 transitions of Fe V, statistical distributions of both log(S/S*) and S/S* are asymmetrical. (In the context of this article, the “true value” S* is the one obtained with the LSF parameter values and *ab initio* values of the E2 transition moments). The distribution of log(S/S*) tends to be skewed to the negative side, while S/S* has a positive skew, meaning that there are more highly deviating values with *S* > S* than with *S* < S*. A somewhat better symmetry is observed for (S/S*)^1/3^. However, I have found that no single function of *S* can produce a normal statistical distribution of results for every transition. For any single function **f**(*S*), a few percent of all calculated transitions have extremely high volatility in the sense that small random variations of the input parameters with normal distributions around the initial values lead to a large number of strongly deviating results. For some transitions, there are too many trials in which the calculated quantity deviates from the initial value **f**(S*) by more than 3*σ*, as compared to the normal distribution. For such highly volatile transitions, which always exist if the same function **f**(S*) is used for all transitions, it is impossible to provide a definitive value of uncertainty in its normal sense, because there is a significant probability that the true value differs from the calculated one by ≥5*σ*. It is usually assumed that such highly volatile transitions are those that are affected by cancellations. This also turns out wrong. Some of the highly volatile transitions indeed have strong cancellation effects, but a significant fraction of them have weak cancellation. Thus, it is necessary to investigate the shape of the distribution function for each calculated transition and choose a function **f**(*S*) that has a statistical distribution closest to normal.

The second note concerns the identification of transitions. Generally, when the Slater parameters are changed, diagonalization of the Hamiltonian results in changes in both eigenvalues and eigenvectors, as well as in the predicted transition wavelengths. Identification of transitions produced by such different calculations poses a serious technical problem, since the level ordering is not necessarily preserved. The method that works well for this purpose, is the eigenvector recognition used in my version of Cowan’s LSF procedure [4], explained in [3]. There is a physical limitation on the applicability of this method, as well as any other method of identification of results produced by different theoretical calculations. Namely, if the atomic models used in the different calculations differ too much (in the context of this article, the atomic model is defined by the set of input parameters), there may be no unique physical relation between the results of these calculations. However, if the differences in the input parameters are sufficiently small, such relation can easily be found by the eigenvector recognition technique. This is especially easy and fast if configuration mixing is small, which is the case for the ground configuration of Fe V, 3d^4^.

The third note concerns the calculated radiative rates, *A*. The small changes of the input parameters cause changes in the calculated wavelengths. The primary calculated quantity is the line strength *S*, Variations of *S* due to small changes of parameters are small, but changes in *A* may be very large, especially for E2 transitions, since *A* is calculated from *S* using relations involving high powers of wavelength. Thus, if one wants to compare the *A* values from different calculations, they should be first rescaled to the same transition wavelengths. In the present work, this rescaling is made for all calculated *A* values; thus, in this work, the relative variations *δA/A* are identical to those of the line strength, *δS/S*.

Before describing the implementation of the method, a brief description of Cowan’s suite of codes is necessary. The suite consists of four separate programs that are run consecutively. The programs are named with Cowan’s initials, “RC”, followed by a letter symbol defining the code category (“N” for the program calculating single-configuration wavefunctions, “G” for the program diagonalizing the matrix of the Hamiltonian and computing the spectrum, “E” for the LSF program). An exception is made for the RCN2 program, which will be explained below. Each program has one or more input files and produces output files, some of which are intended to be input files for the next program in the chain. Thus, RCN has an input file that defines the configurations to be included in the calculation, as well as several parameters having technical purposes, such as the tolerance for iterations in the self-consistent field calculations. As output, RCN produces a binary file defining the computed wavefunctions, which is then used by the next program, RCN2, as an input file. RCN2 also has an additional input (text) file that can be modified by the user. This file contains definitions of some adjustable parameters, such as scaling factors for Slater parameters. Input files that can be modified by the user have in their names letters “in” followed by the code symbol and sometimes by numerals defining the code version. Thus, the input file for RCG version 11, produced by RCN2, is named “ing11.” This file contains all parameters needed for the construction of the Hamiltonian matrix, such as Slater and CI parameters denoted below as ***P***), and the multipole transition matrix elements. The program RCG (version 11), in turn, produces output files “outg11” and “outgine.” The first of them contains the calculated spectrum (eigenvalues, eigenvectors, wavelengths, and transition probabilities), and the second one is an input file for the RCE program. In addition to these text files, RCG produces a binary file with additional input data for RCE, which, among other things, contains the matrix of the partial derivatives of parameters ***P*** over eigenvalues ***E***, [*∂****P****/∂****E***]. This matrix is used by RCE to adjust ***P*** in an iterative way so as to produce the minimum standard deviation of ***E*** from experimental energies ***E***_exp_. The standard deviation of the fitted parameters is then computed as

(1) $$\Delta P_{\text{LSF}}=\partial P/\partial E\;(E_{\exp}-E).$$

This organization of the codes allows the user to make either ab initio or semi-empirically adjusted calculations. In particular, the least-squares-fitted parameters ***P***_LSF_ produced by RCE can be easily transferred to the input file of RCG, ing11, to calculate the semi-empirically adjusted spectrum.

The procedure implemented here is as follows.

1. The main directory of the calculation is set up with the input and output files for the Cowan-code calculation with LSF. The LSF for the even parity of Fe V was made earlier as described in [5].
2. The control code creates a separate subdirectory for each random trial and sets up all files necessary for calculations in each of these subdirectories. The maximum number of random trials used in this test implementation was 10,000, so there were 10,000 sub-directories.
3. The control code reads the input file for the matrix-diagonalization code RCG (ing11) from the main directory and prepares sets of randomly varied E2 matrix elements for each trial. The random variations are implemented using a standard random number generation routine (producing uniformly distributed random floating-point numbers in the interval between 0 and 1), converted to normally distributed numbers using the Box–Miller transformation [7] (see Section 3.2). The centers of these normal distributions are set to be equal to the initial values of the input parameters. For the E2 matrix elements, the width of the normal distribution of the generated random numbers was arbitrarily set to 1% of the initial parameter value. Using these generated sets of randomly varied E2 matrix elements, the control code creates input files for the RCG code in each trial subdirectory. At this step, all Slater and CI parameters are kept the same as in the main directory.
4. The control code reads the output file of RCG (outg11) and other auxiliary files from the main directory (including ing11), reads the output file of the LSF code RCE (oute), finds the data block corresponding to the last LSF iteration, and reads the fitted parameter values (Slater and CI) and their *σ*. It also reads the eigenvectors resulting from the last LSF iteration. In my version of Cowan’s RCE code, the eigenvectors produced by LSF are saved in an additional output file named rceout.
5. The control code identifies the eigenvectors produced by RCG with those produced by RCE (which are nearly identical, since the preliminary RCG calculation was made in the main directory using the parameter values from LSF), and thus establishes correspondence between the initial calculated eigenvalues and experimental energies.
6. The control code continues reading the part of the outg11 file from the main directory containing the transition data (initial and final energy levels, wavelengths, *A*-values, and cancellation factors).
7. In each trial subdirectory, the control code randomly varies the Slater and CI parameters using the same procedure as for the E2 matrix elements, except that the widths of normal statistical distributions are set to be equal to the corresponding *σ* of the LSF. The parameters that were linked together at a fixed ratio in the LSF are varied in the same linked manner. Namely, their scaling factor is varied, but the ratios within each linked group remain fixed. For parameters that were fixed (not varied) in the LSF, the width of the normal distribution of the varied values was arbitrarily set to 2% of the parameter value. The varied parameters are substituted into the ing11 file (input file for RCG) prepared earlier in each subdirectory in step 3.
8. In each trial subdirectory, the control code runs RCG, reads the resulting outg11 file containing new eigenvectors and sets of transition data, identifies the new eigenvectors with the old ones, kept in memory from step 5, and, for each transition, rescales the new *A*-values to the experimental (Ritz) wavelengths, and appends the statistics data.
9. The accumulated statistics data are processed and results are printed to an output file.

The control code was written in Perl programming language. It has about 1,000 lines of code and uses several external Perl utility codes developed earlier and included with the Cowan code package [4]. These utilities include, for example, the eigenvector recognition routine and have about 3,000 lines of code. They were optimized for speed for the present purpose. With 10,000 random trials, the code execution takes about a few hours on a moderately powerful personal computer with 16 Gb of memory. Most of this time is taken by execution of RCG on each trial.

## 3. Results and Discussion

The 3d^4^ configuration of Fe V consists of 34 energy levels spanning the range from zero to 94,000 cm^−1^, all of which are experimentally known with uncertainties ranging from 0.3 cm^−1^ to 1.5 cm^−1^ [5]. My calculations with Cowan’s codes yield 232 M1 transitions and 358 E2 transitions between these levels. For each of these transitions, the Monte Carlo method described in Section 2 produced a set of up to 10,000 *A*-values, providing a basis for statistical analysis. Results of this analysis are described below.

### 3.1. Input Parameters

As described in [5], the calculation of the even parity complex of Fe V included eight configurations, 3d^4^, 3d^3^(4s + 5s + 4d + 5d), and 3d^2^(4s^2^ + 4s4d + 4d^2^). Thus, there were 38 non-zero E2 matrix elements for transitions between these configurations, 86 Slater parameters (average energy *E*_av_, spin-orbit parameters *ς*_3d_ and *ς*_4d_, direct and exchange Coulomb interaction parameters *F*^2,4^(*n*d,*n*′d) and *G*^0,2,4^(*nl*,*n*′*l*′), respectively, and effective parameters *α*_3d_, *β*_3d_, and *T*_3d_; those are coefficients of Casimir operators representing many-body effects in shells with equivalent electrons, see Cowan’s book [4], section 16-7), and 61 CI parameters. In the LSF, many of these parameters were grouped with fixed ratios within each group. In particular, all CI parameters were linked in one group. In total, there were 15 such groups, which included 121 parameters, nine parameters were allowed to vary independently, and seven parameters were fixed in the LSF (note that these were allowed to vary with a relative *σ* of 2% in the Monte Carlo trials). Thus, in the random trials there were 69 variable parameters, 38 of which affected only E2 transitions.

Calculations of M1 transitions are intrinsically more accurate, because they involve only linear combinations of amplitudes of the eigenvector components of the lower and upper levels with coefficients that are functions of quantum numbers of the basis states. Thus, the calculated M1 *S*-values change only due to changes in the eigenvector component amplitudes. On the other hand, the E2 *S*-values are additionally affected by the (unknown) uncertainties in the E2 transition integrals, which depend on the accuracy of the radial wavefunctions. In the present investigation, these uncertainties were arbitrarily assumed to be equal to 1%, just so that transitions strongly sensitive to these uncertainties would be detected. The statistical distribution of the random trials was preliminary tested with a million trials and was found to be indeed very close to normal with the given variance (described above in Section 2).

The LSF parameters used as initial input for Monte Carlo trials are listed in Table 1. They were obtained in the work reported in [5]. The acronym ‘HFR’ appearing in the last two columns of Table 1 means ‘Hartree–Fock-Relativistic’ and is commonly used to denote the approximation used in *ab initio* calculations with Cowan’s codes, the Hartree–Fock method with relativistic corrections. It should be noted that the standard deviation of the eigenvalues produced by the LSF from experimental energies is 117 cm^−1^ for all 220 experimentally known even levels and 41 cm^−1^ for the 34 levels of 3d^4^. Thus, it is a fairly good fitting with well-defined parameters. As can be seen in Table 1, the fractional uncertainties of ***P***_LSF_ are in the range between a small fraction of a percent for *E*_av_ and about 10% for *α*_3d_ and *β*_3d_, except for *β*_3d_(3d^5^5s), which had a larger uncertainty.

### 3.2. Statistical Distributions of A-Values Obtained with 1000 Random Trials

**Figure 1:**
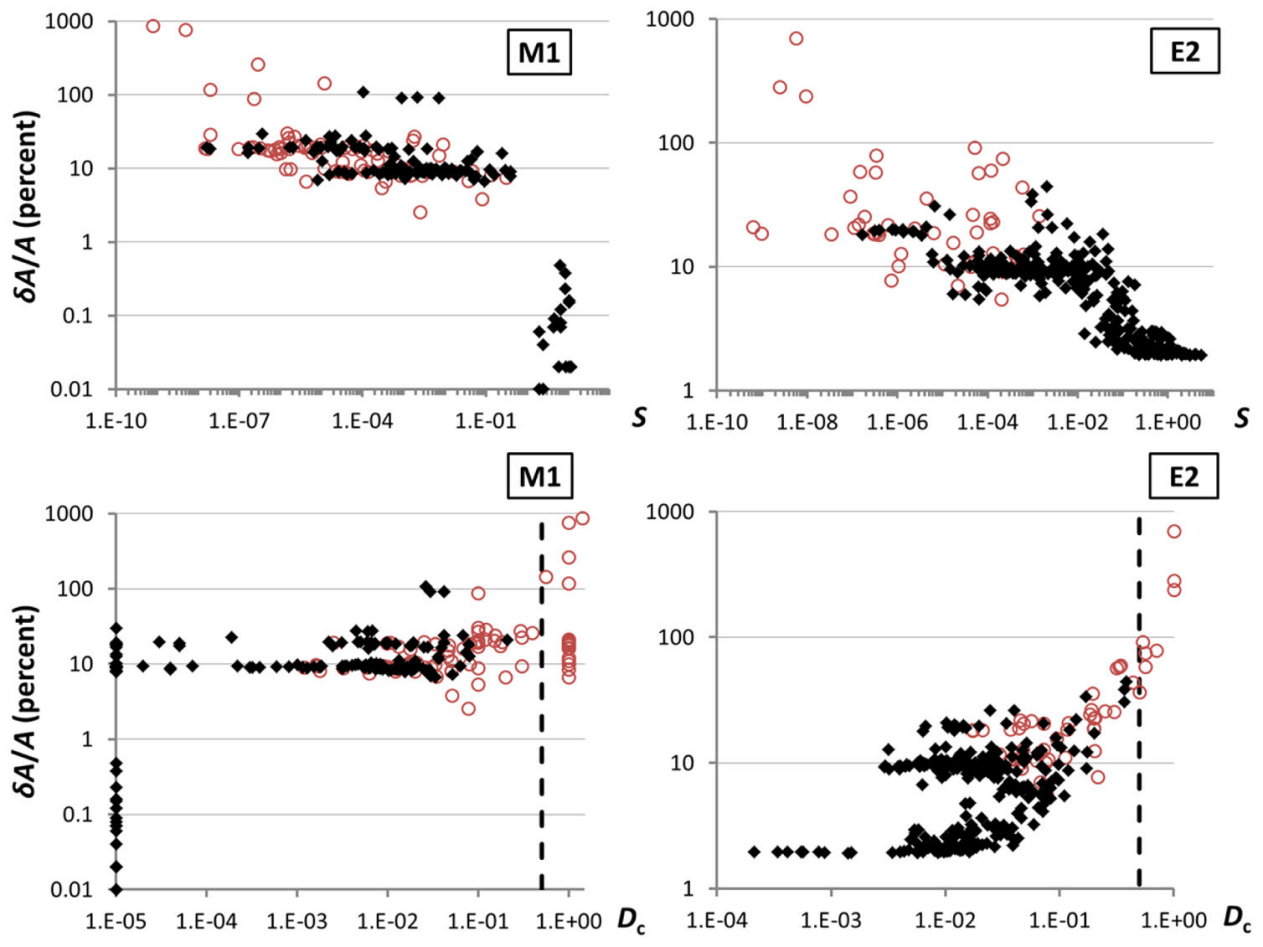
Relative **s**tandard deviations *δA*/*A* (percent) of Monte Carlo trial data for M1 (**left**) and E2 (**right**) transitions of Fe V *versus* line strength *S* (**top**) and *versus* degree of cancellation *D*_c_ = *δ*_CF_/|CF| (**bottom**). Full symbols—transitions with weak cancellation, |CF| ≥ 0.01; open symbols—transitions with strong cancellation, |CF| < 0.01. Vertical dashed lines in the bottom part show the boundary between regions of significant and insignificant cancellation, *D*_c_ > 0.5 and *D*_c_ < 0.5, respectively.

Let us start with something familiar to atomic physicists, at least to some extent. Namely, let us consider how the standard deviations *σ* of *A* values behave depending on the line strength *S*. This dependence is presented, separately for M1 and E2 transitions, in the upper half of Figure 1. At first glance, the qualitative behavior of these plots confirms the general trend observed by many researchers and traditionally used to estimate the uncertainties of *A* values in different ranges of line strength (see, for example, [3,5]). Namely, for strong transitions the *A* values are well defined, while for weaker transitions uncertainties grow rapidly with decreasing *S*. However, these plots differ from traditional comparison plots [3,5] in that each data point represents not a single comparison between two calculations, but a standard deviation over 1,000 different calculations.

For strong M1 transitions with *S* > 1, the calculations are especially stable, giving *σ* over 1,000 trials well below 1%. I was tempted to use the word “accurate” in this statement. However, one should remember that this discussion is about statistical distributions of results of one particular computational model. The accuracy of this model is necessarily limited by the approximations used. Thus, the *uncertainties* of its results must be greater than the standard deviations discussed here. This point will be further discussed in the following subsections.

If we look closer at the layout of the data points in the upper part of Figure 1, we can see that, in the same region of line strengths, the *σ* of resulting *A* values strongly differ from each other. For example, for M1 transitions with *S* = (0.001–0.01), most of the data points are clustered around *σ* ≈ 10%. However, there are many data points having *σ* greater or smaller than that by up to an order of magnitude. In the standard method of evaluation of uncertainties by comparing a few different calculations with each other, the apparent outliers with too large deviations would be assigned uncertainties much greater than the average for a given line strength. However, because of low statistics in such comparisons, there is a big chance of occasional coincidences, so some of such highly volatile transitions would not be detected. On the other hand, for some transitions that happen to be much more stable than the others with a similar *S*, uncertainties would be greatly overestimated.

Large deviations of calculated *A* values for some transitions are usually explained by strong cancellation effects. In Cowan’s codes, these effects are characterized by the so-called cancellation factor (CF) defined as follows. Calculation of the line strength of a transition is made by summation of contributions of different sign involving eigenvector components of wavefunctions of the lower and upper states. All positive and negative contributions are summed separately in partial sums *S*^+^ and *S*^−^, so that the line strength is given by *S* = *S*^+^ + *S*^−^. Then CF is computed as CF = (*S*^+^ − |*S*^−^|)/(*S*^+^ + |*S*^−^|). Thus, CF ∈ [−1, 1], and “strong cancellation” occurs when |CF| is close to zero.

I tried to find a correlation between the strong cancellation and the high volatility of *A*-values by separating transitions with low cancellation factor |CF| < 0.01 (red circles in Figure 1) from those with large CF, |CF| ≥ 0.01 (full symbols). The upper part of Figure 1 does not reveal any strong dependence of relative deviations *δA*/*A* on |CF|. In general, transitions with low |CF| tend to be weaker, so the red circles are clustered in the regions of smaller *S*. However, in the regions of similar *S*, the red circles (strong cancellation) are intermingled with full symbols (weak cancellation). Thus, the small cancellation factor per se cannot serve as a definitive indicator of bad quality of calculations.

I attempted to investigate the cancellation effects in more detail. For each transition, each trial calculation produces a somewhat different CF. The CF values appear to be distributed normally around the initial values for most of transitions. Only for about 10% of all transitions distributions of CF are far from normal. The widths (*i.e.*, standard deviations) of these distributions, *δ*_CF_, are very different for different transitions, varying from 10^−18^ to 0.07. To better characterize the cancellation effects, I introduce a new quantity, degree of cancellation *D*_c_ = *δ*_CF_/|CF|. The cancellation effects are truly significant if *D*_c_ is greater than 0.5. In such cases, some trial calculations produce a positive CF, others negative. Dependence of relative deviations *δA*/*A* on *D*_c_ is plotted in the lower part of Figure 1. For E2 transitions, one can see that, indeed, all transitions with *δA*/*A* > 100% have *D*_c_ > 0.5. However, for M1 transitions it is not so. There are some transitions with a rather small degree of cancellation *D*_c_ having *δA*/*A* greater or close to 100%. At the same time, many transitions with truly strong cancellation (*D*_c_ > 0.5) have unexpectedly small deviations *δA*/*A*. Thus, even this statistically augmented approach does not allow one to definitively differentiate between “good” and “bad” results.

**Figure 2:**
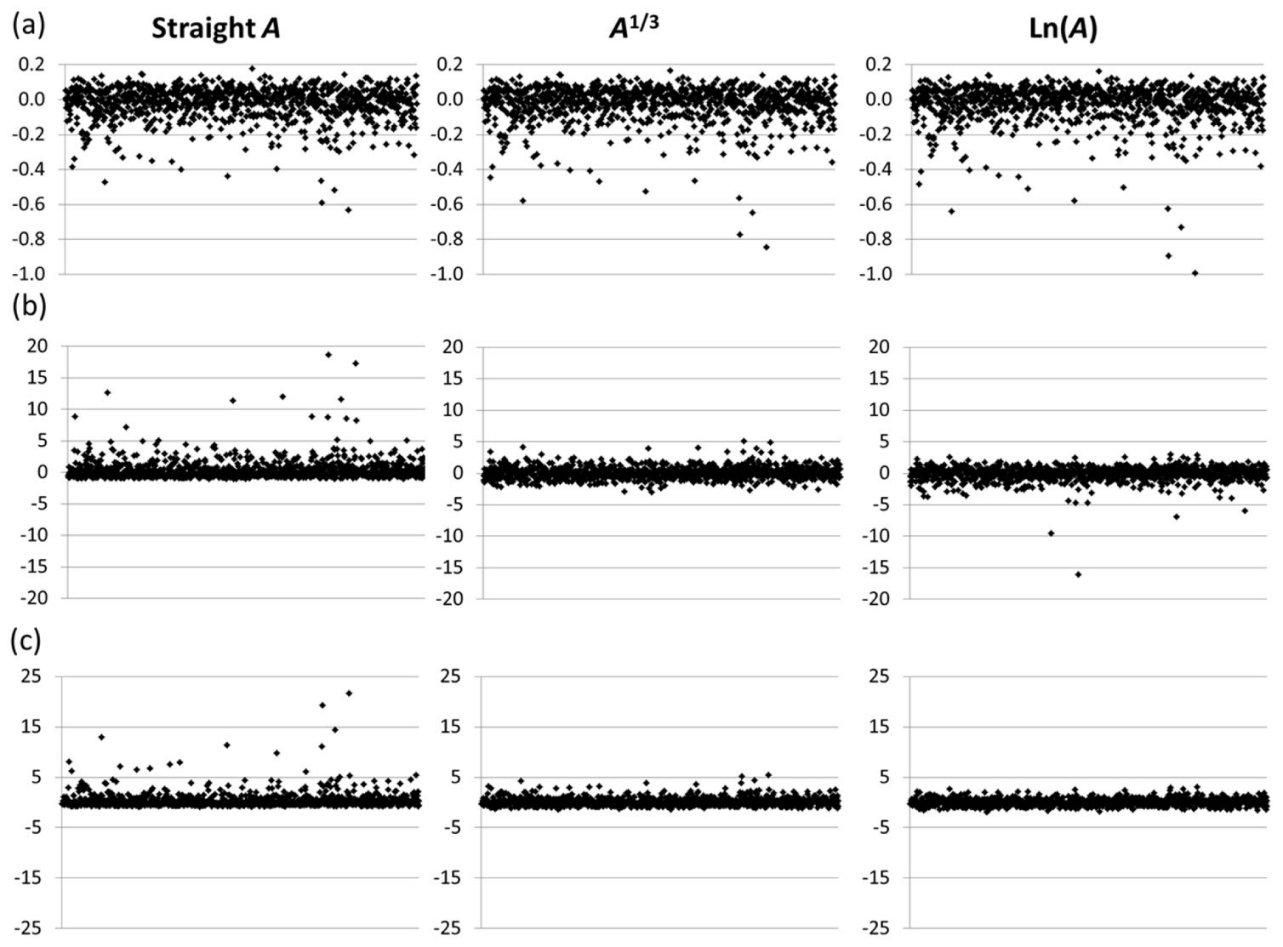
Relative deviations *δ***f**/**f** of Monte Carlo trial data for three considered transition-rate functions, straight *A* (**f** = *A*), power of 1/3 (**f** = *A*^1/3^), and logarithm (**f** = ln(*A*)) for three typical E1-forbidden transitions within the ground configuration 3d^4^ of Fe V. (**a**) ^3^F2_2_–^3^F1_4_ E2, (26,760.7–62,238.0) cm^−1^, *λ* = 2,817.87 Å, *A* = 7.18 × 10^−3^ s^−1^, |CF| = 0.14(3); (**b**) ^5^D_2_–^3^F2_2_E2, (417.5–26,760.7) cm^−1^, *λ* = 3,794.9 Å, *A* = 2.92 × 10^−9^ s^−1^, |CF| = 0.00001(1); (**c**) ^3^P2_2_–^3^G_3_ M1, (26,468.2–29,817.1) cm^−1^, *λ* = 29,861.4 Å, *A* = 1.42 × 10^−5^ s^−1^, |CF| = 0.89(2).

Now let us take a look at statistical distributions of results for individual transitions, something that was never considered before in the literature. These distributions are shown in Figure 2 for three typical transitions. In this figure, the quantity on the vertical axis is the relative deviation *δ***f**/**f** (unlike Figure 1, where *δA*/*A* is given as percentage, here **f** = **f**(*A*) is a certain function of *A*, and the quantity on the vertical axis is *δ***f**/**f** = **f**(*A*)/**f**(A*) − 1). On the horizontal axes, it is just the trial number from 1 to 1,000. Each panel (a–c) corresponds to a different transition (specified in the figure caption). The three columns of figures represent results obtained for three different functions of *A*, **f** = *A* (“straight *A*”), **f** = *A*^1/3^, and **f** = ln(*A*).

One can see from Figure 2 that statistical distributions of results obtained for all presented transitions are asymmetrical and have significant numbers of far outliers, the number and location of which depends on the function used. (Hereafter, “outlier” means a data point deviating from the mean by more than one *σ*, and “far outlier” means a datum deviating from the mean by more than 3*σ*). For each transition, some functions appear to give “better” statistical distributions than others, both in terms of symmetry and the number of outliers. Thus, for transition (b), the function **f** = *A*^1/3^ appears to give a fairly symmetrical distribution with a relatively small number of high deviations, while for transition (c), the best of the three functions is **f** = ln(*A*). However, for transition (a), although the “straight *A*” distribution appears to have the lowest number and size of high deviations, none of the three functions have a symmetrical distribution.

To investigate how the different functions **f**(*A*) fair in regards to the closeness of their statistical distributions to normal, I counted the numbers of trials producing relative deviations *δ***f**/**f** > *nσ*, where *n* is an integer number from 0 to +5 and similar numbers for negative *δ***f**/**f** less than −*nσ*. The quantity defining the fractional numbers of these counts is denoted *N*(*n*) below. These counts were co-added for all 590 investigated transitions and compared to the counts predicted with the cumulative normal distribution function, *N*_norm_(*n*). For reference purposes, the values of *N*_norm_(*n*) are given in Table 2.

One can see from Table 2 that, with 1,000 trials made for each transition, with the normal distribution, the probability of obtaining a result deviating from the mean by >5*σ* is negligible. However, if counts for all transitions are co-added, this gives a total number of trials of 590,000, and the probability of having at least one outlier deviating by more than ±5*σ* should be about 0.3 for the normal statistical distribution.

**Figure 3:**
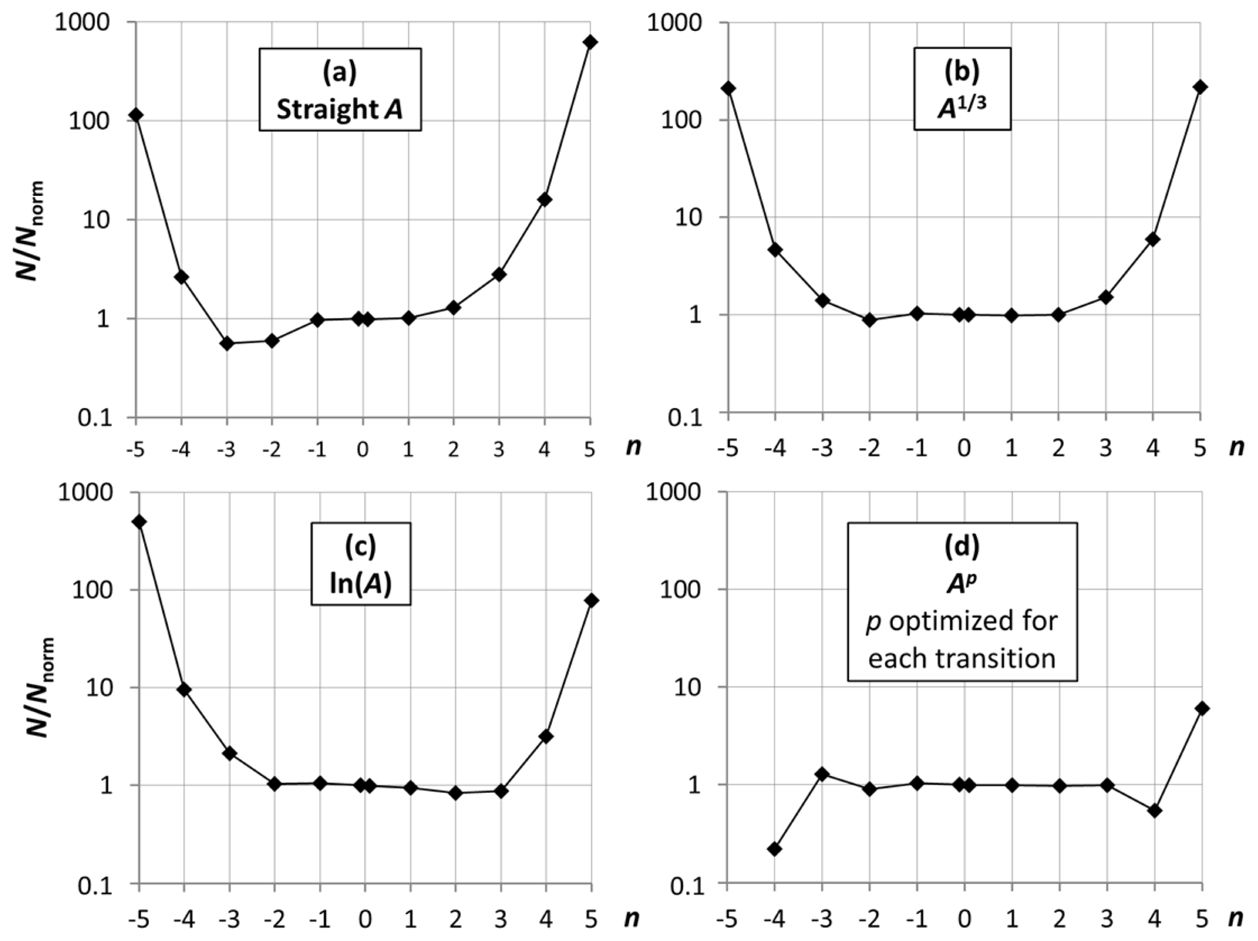
Ratios of fractional number of counts *N*(*n*) of Monte Carlo trial data to those of the normal distribution *N*_norm_(*n*) for three global transition-rate functions, (**a**) straight *A* (**f** = *A*); (**b**) power of 1/3 (**f** = *A*^1/3^); and (**c**) logarithm (**f** = ln(*A*)) for all 590 M1 and E2 transitions of Fe V; Panel (**d**) shows the same ratios for **f** = *A^p^*, where *p* is optimized individually for each transition.

The ratios of *N*(*n*)/*N*_norm_(*n*) obtained with different functions **f**(*A*) are depicted in Figure 3. First let us consider the three functions discussed above, **f** = *A* (panel a), **f** = *A*^1/3^ (panel b), and **f** = ln(*A*) (panel c). Panel (a) shows that the distribution of straight *A* values is skewed to the positive side, meaning that there are more trials in which *δA*/*A* > 0 than those with *δA*/*A* < 0. The distribution of **f** = ln(*A*), shown in panel (c), is similarly skewed to the negative side. The distribution of **f** = *A*^1/3^, shown in panel (b), is fairly symmetrical (on average; one should keep in mind that these plots are drawn for the overall statistics of all transitions). However, for all these functions the number of far outliers (those with |*n*| > 4) is much greater than one would expect for a normal distribution.

I attempted to divide all considered transitions in three groups based on the “best” function **f**(*A*) out of the three ones considered above. The “straight *A*” function turned out to be the best (of the three) for about 14% of all transitions (10% of M1 transitions and 17% of E2 transitions). The function **f** = *A*^1/3^ is the best one for 47% of all transitions (51% of M1 and 45% of E2), and **f** = ln(A) is the best for 39% of all transitions (40% of M1 and 38% of E2). Thus, neither of these functions provides the best statistics for a significant majority of transitions.

Furthermore, even when the best of the three functions is used for each transition, the number of far outliers is still much greater than for the normal distribution. No apparent connection was found between the choice of the best function and physical characteristics of a transition, such as the type (M1 or E2), line strength, wavelength, energy levels involved, and the degree of cancellation.

Thus, the logarithmic function, which I earlier assumed (in [3,5] and several other papers) to provide statistics close to normal, in fact does so only for less than half of all transitions (at least it is so for the M1 and E2 transitions considered here).

In retrospective, the failure to find one or a few simple functions of *A* having good statistics for all transitions should not be surprising. The *A* values are numerically calculated in a procedure involving a matrix diagonalization, so it is obvious that they are complicated functions of the many input parameters. There is no reason to believe that there exists a single simple function of *A* providing a normal statistical distribution for all transitions.

However, it turned out possible to find the best function of a general type described by the Box–Cox transformation function [7]:

(2) $$f=\left\{\begin{array}{c} \left[\frac{{\left(A/A^{*}\right)}^{p}-1}{p}\right],p\neq 0\\ \ln(A/A^{*}),p=0 \end{array}\right\}.$$

**Figure 4:**
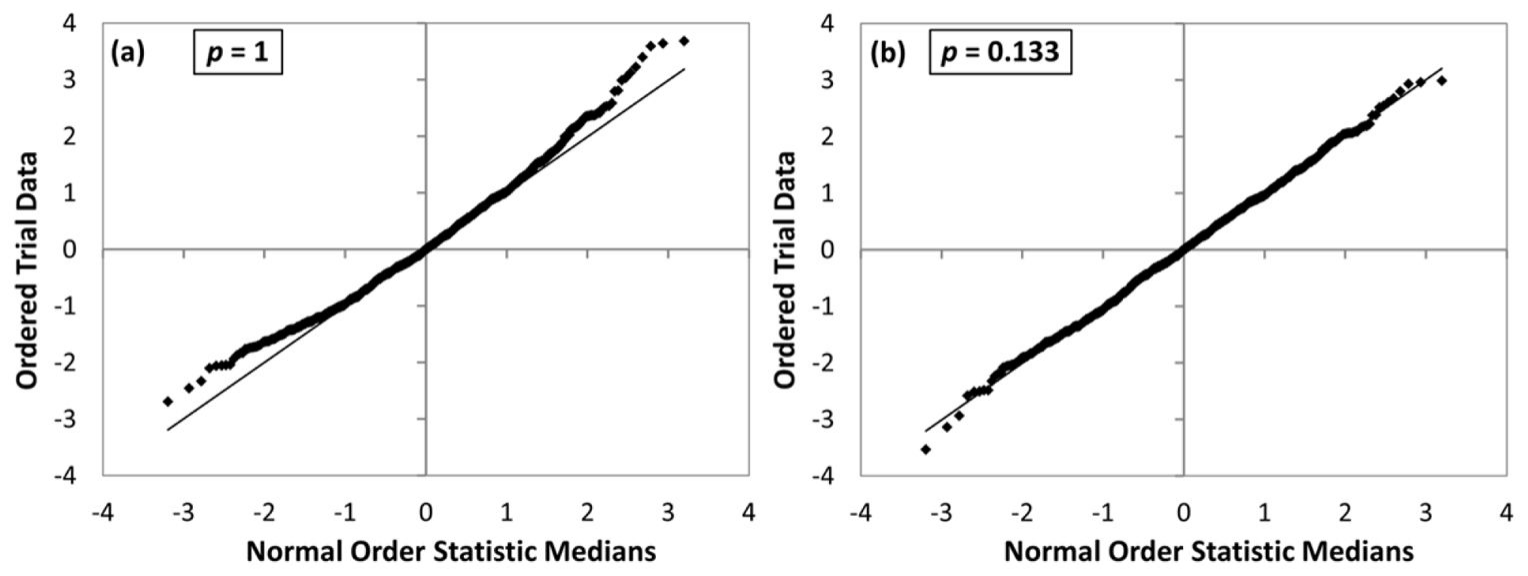
Examples of normal probability plots for one transition (see text). (**a**) The plot with no transformation of *A* values (*p* = 1) shows departure of the statistics from the normal distribution; (**b**) The plot with the optimal Box–Cox transformation parameter (*p* = 0.133) shows that the statistics are close to normal.

Although the Box–Cox transformation is defined piecewise, it is a smooth function of *p*, *i.e.*, it does not have any singularity at *p* = 0. In applied statistics, there is a well-known technique for finding the optimal values of the parameter *p* of the Box–Cox transformation providing statistics closest to normal. This technique utilizes the so-called *normal probability plots* [8]. In such plots, the trial data are plotted against a theoretical normal distribution in such a way that the points should form an approximate straight line. Departures from this straight line indicate departures from normality. Examples of such normality plots are given in Figure 4 for the ^5^D_1_–^1^S1_0_ M1 transition at 826.53 Å. Panel (a) was drawn for the “straight *A*” transformation function (*p* = 1 means no transformation), while panel (b) presents the transformed trial data with the optimal value of *p* = 0.133. It can be seen from panel (a) that the raw *A* data significantly deviate from the normal statistical distribution, while the near linearity of the plot in panel (b) shows that the data transformed with *p* = 0.133 are nearly normally distributed. The quantity on the vertical axis of Figure 4 is the normalized response value (**f** − f*)/*σ*, where *σ* is the standard deviation of **f** and the starred symbols mean the reference values obtained in the original LSF fitting. The trial data are ordered in the order of increasing response value. For each data point *i* (1 ≤ *i* ≤ *n*, where *n* is the number of trials, 1,000 in this case), the corresponding value of the normal order statistic median (given on the horizontal axis) is calculated with the function *N_i_* = *G*(*U_i_*), where *G* is the inverse of the cumulative normal distribution function (probability that its argument is less than or equal to some value), and *U_i_* are the uniform order statistic medians defined as follows:

(3) $$\begin{array}{c} U_{n}=0.5^{1/n}\\ U_{i}=(i-0.3175)/(n+0.365)\;\;\;\text{for}\;i=2,3,\ldots ,n-1\\ U_{i}=1-U_{n}\;\text{for}\;i=1 \end{array}$$

**Figure 5:**
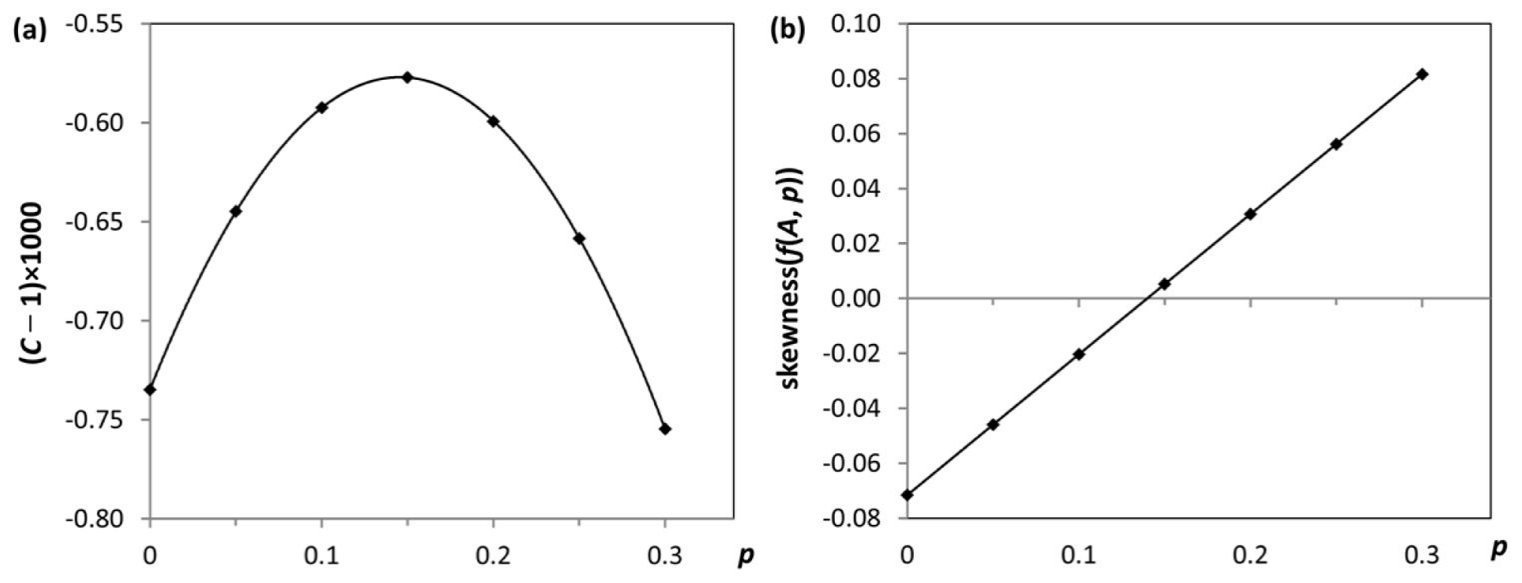
Two methods of finding the optimal parameter *p* of the Box–Cox transformation: (**a**) By maximizing the correlation coefficient *C* of the normal probability plot; (**b**) By finding the value of *p* yielding a zero *skewness* of the distribution of **f**(*A*, *p*).

The optimal value of *p* is found by maximizing the correlation coefficient of the normal probability plot. This is illustrated in Figure 5a, where the correlation coefficient *C*(*p*) is plotted against *p* (this plot uses the trial data for the same transition as in Figure 4). The shape of the function *C*(*p*) turned out to look similar for all studied transitions, but the position of its maximum varies in a wide range of *p*.

An alternate, somewhat simpler method of finding the optimal Box–Cox transformation is to find the value of *p* at which the response data have zero *skewness*, which is defined as follows:

(4) $$\mathrm{skewness}=\frac{\sum_{i=1}^{n}{\left(x_{i}-\bar{x}\right)}^{3}}{(n-1)\sigma^{3}}$$

For a normal distribution, the *skewness* is zero. However, in general the zero value of *skewness* does not guarantee that the distribution is close to normal; it only ensures that it is symmetric. Another parameter characterizing the shape of a distribution function is *kurtosis*. For this quantity, I use the definition that is usually referred to as “excess kurtosis”:

(5) $$\mathrm{kurtosis}=\frac{\sum_{i=1}^{n}{\left(x_{i}-\bar{x}\right)}^{4}}{(n-1)\sigma^{4}}-3$$

For a normal distribution, thus defined *kurtosis* is zero. Positive values indicate a “peaked” distribution, while negative values indicate a “flat” distribution.

For transitions considered in this work, it turned out that, for the optimal values of *p* that minimize *skewness*, the corresponding values of *kurtosis* were in the range −0.7 to +3.3 with an average of −0.04 and a standard deviation of 0.23. For all considered transitions, thus found optimal values of *p* are close to those found by maximizing the correlation coefficient of the normal probability plot. Both methods of optimizing the parameter *p* are illustrated in Figure 5.

As noted above, the two methods of optimizing the Box–Cox transformation work almost equally well for the considered transitions. However, to avoid ambiguity, hereafter, when mentioning the optimal parameter *p*, I will always mean the results obtained with the first method, *i.e.*, maximizing the correlation coefficient of the normal probability plot. The overall statistic obtained with parameters *p* optimized individually for each transition is shown in panel (d) of Figure 3. Considering the leftmost and rightmost data points in this plot, one should keep in mind that the trial counts for these points are very small. In particular, the *n* = 5 point represents the only case (out of 590,000) in which *δ***f**/**f** exceeded 5. Thus, the apparent deviations from unity for |*n*| ≥ 4 seem to be explained by statistical noise. However, further analysis with larger statistics indicates that this is not true (see Section 3.3).

**Figure 6:**
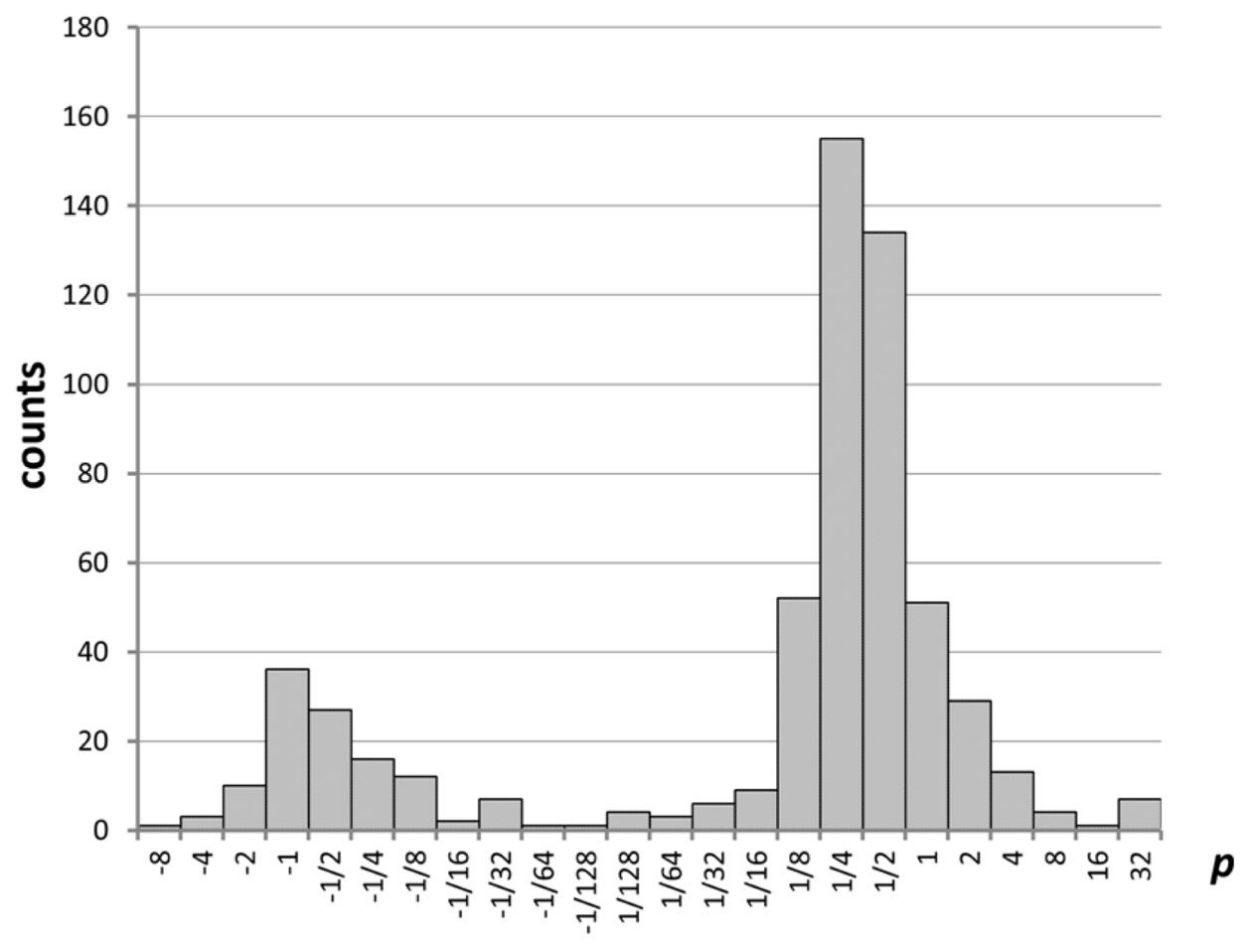
Distribution of optimal values of the Box–Cox transformation parameter *p* obtained in a 1,000-trials run. Intervals on the horizontal axis are logarithmically equal, except for the interval encompassing the zero value. The vertical axis is the number of transitions with *p* close to the value below the horizontal axis (see text).

The optimal values of *p* found for the considered transitions of Fe V span a wide range from about −8 to +34 (a few transitions had extremely large values *p* > 100). The histogram showing the distribution of optimal values of *p* (excluding the abnormal ones with extremely large *p*) is shown in Figure 6. In this figure, the counts plotted on the vertical axis correspond to the number of transitions having the value of *p* between the one shown below each column and the one below an adjacent column. For example, for positive *p*, the value shown for *p* = 1 is the number of transitions with 1 ≤ *p* < 2; the value for *p* = 2 is the number of transitions with 2 ≤ *p* < 3, *etc.* Similarly, for negative *p*, the value shown for *p* = −1 is the number of transitions with −2 < *p* ≤ 1; the value for *p* = −2 is the number of transitions with −3 < *p* ≤ 2, *etc.* The point labeled *p* = 1/128 is an exception, because the corresponding interval encompasses the *p* = 0 value.

The distribution of optimal powers *p* has two distinct peaks, a greater one at *p* ≈ 0.3 and a smaller one near *p* ≈ −0.7. However, there is no peak near *p* = 0, indicating that there are very few transitions for which the logarithmic transformation produces statistics close to normal.

### 3.3. Results with Larger Statistics

**Figure 7:**
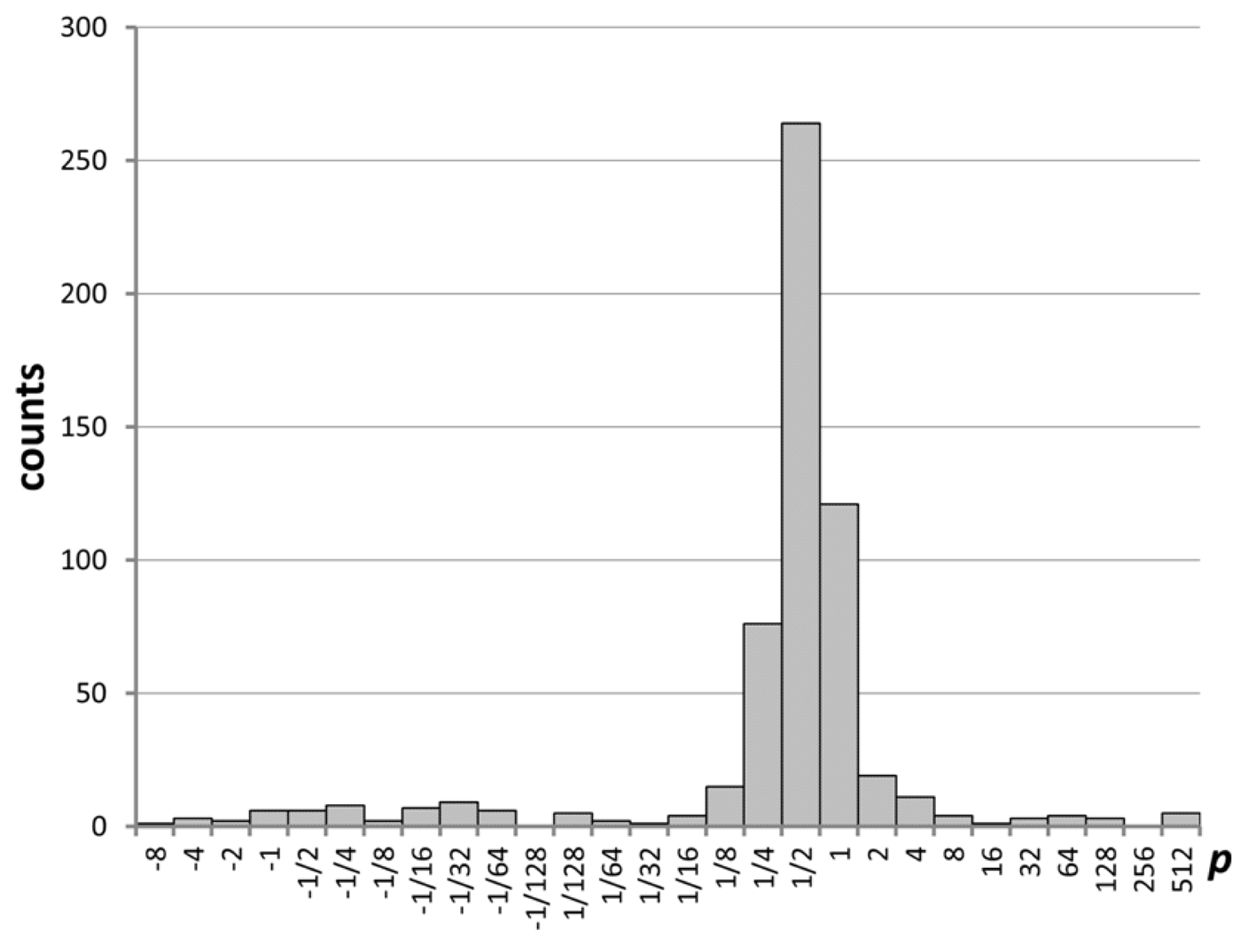
Distribution of optimal values of the Box–Cox transformation parameter *p* obtained in a 10,000-trials run.

After making several runs with 1,000 trials each, I noticed that for many transitions the optimal *p* values differ strongly from one run to another. This indicated that 1,000 trials give insufficient statistics for accurate determination of *p*. This is due to the fact that the optimal transformation strongly depends on the far outliers (*i.e.*, those values of *A* that strongly deviate from the initial ones). As discussed above, 1,000 trials result in a relatively small number of results deviating from the initial values by more than 4*σ*. Therefore, I made an extended run with 10,000 trials. Combining the results of this run with those from four 1,000-trials runs allowed me to derive more accurate values of *p* and estimate their uncertainties as standard deviations of the mean over several runs. The distribution of these final values of *p* among all transitions is shown in Figure 7. One can see that it differs significantly from Figure 6 obtained from one 1,000-trials run. Now the histogram has only one major peak near *p* = 0.5.

**Figure 8:**
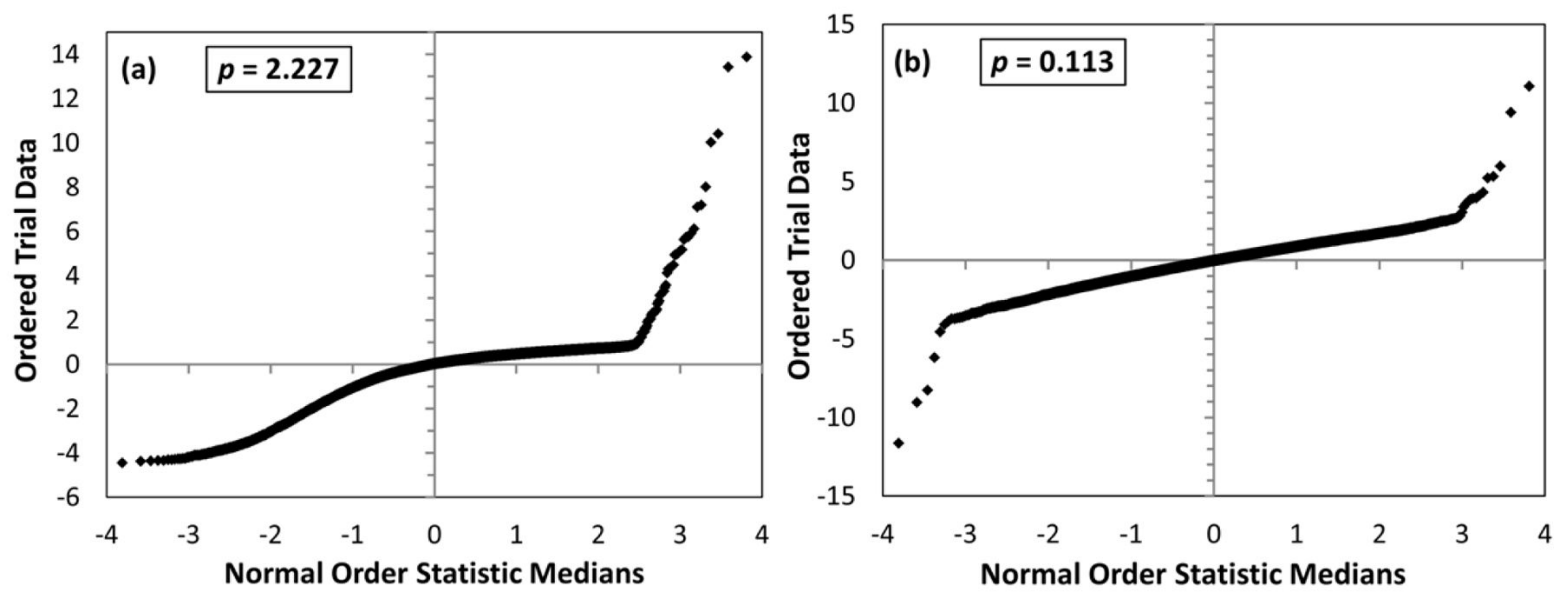
Examples of normal probability plots for abnormal transitions (see text). (**a**) ^5^D_2_–^3^P2_2_ M1 transition, *λ* = 3,837.58 Å, *A* = 4 × 10^−5^ s^−1^, |CF| = 2(4) × 10^−6^; (**b**) ^1^G2_4_–^3^D_3_ E2 transition, *λ* = 2.252 μm, *A* = 1.56 × 10^−17^ s^−1^, |CF| = 0.0824(13). In both panels, the data are plotted for an optimal Box–Cox transformation with the parameter *p* shown in boxes.

Larger statistics revealed that for a few transitions the optimal Box–Cox transformation is far from normal. Examples of normal probability plots for some of such “abnormal” transitions are given in Figure 8. All such abnormal transitions are extremely weak. A few transitions required very large values of *p* for an optimal transformation. Thus, five transitions have *p* = (50–75), seven have *p* = (100–600), and one has *p* = 2,621. The latter is the ^3^P2_1_–^3^P2_2_ M1 transition at *λ* = 66,872 Å with *A* = 4.541 × 10^−2^ s^−1^.

**Figure 9:**
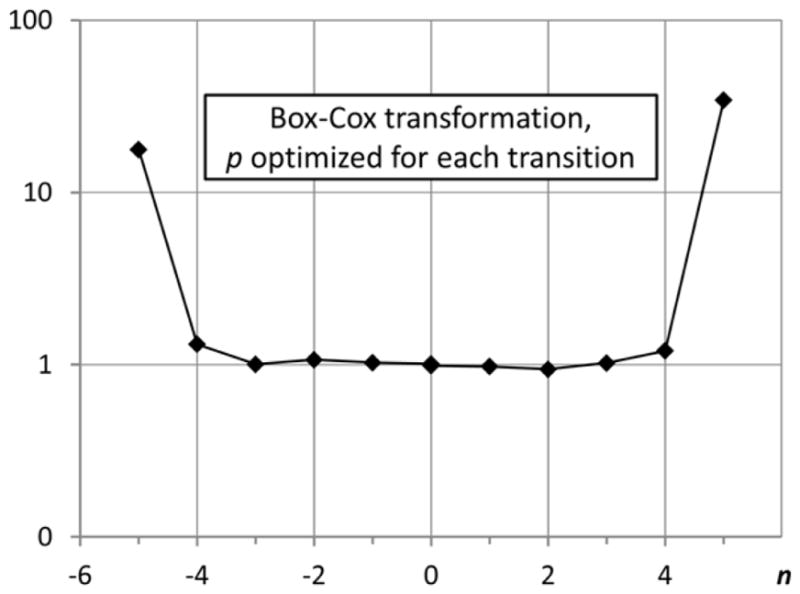
Similar to Figure 3d, with larger statistics (10,000 trials) and with optimal Box–Cox transformations applied to all transitions.

When optimal Box–Cox transformations are applied to all transitions, the overall comparison of statistical distributions with normal (similar to the plot given in Figure 3d) looks as shown in Figure 9.

One can see from Figure 9 that the Box–Cox transformation is unable to rectify statistical distributions for all transitions. In the particular case of Fe V transitions considered here, all far outliers accounted for in Figure 9 in the statistics with *n* = ±5 (more than 5*σ*) correspond to extremely weak transitions.

### 3.4. Uncertainties of A-Values

Strictly speaking, the standard deviations of optimally transformed *A* values obtained with the described method do not yield dependable estimates of uncertainties. Rather than that, they represent lower limits of those uncertainties. Additional contributions to the uncertainties come from the approximations made in the atomic model used. When calculations are made with one theoretical atomic code, they should include a study of convergence effects in regards to systematical improvement of the atomic model, such as a growing number of included layers of orbitals with increasing quantum numbers. Furthermore, a realistic estimate of uncertainties should involve both internal estimations based on the sensitivity of each transition to the variation of input atomic parameters and from comparisons with results obtained with other theoretical models.

None of these uncertainty contributions are considered in the present work. Nevertheless, the obtained optimal transformation parameters *p* and relative standard deviations *σ_f_* of transformed *A* values **f**(*A*, *p*) may be useful for further investigations. Therefore, they are given in Table 3 for each transition included previously in [5]. Most of these transitions have a branching fraction greater than 0.001. Transitions having significant contributions of both M1 and E2 type (greater than 0.01) are considered to be of a mixed type M1 + E2. For such mixed transitions, the shapes of the statistical distributions were investigated for the sum *A*^M1^ + *A*^E2^, and the optimal values of *p* were found for these sums.

A rough estimate of relative *σ* for *A* values (*σ_A_*, standard deviation of *δA*/*A*) is given in Table 3 along with the standard deviations of transformed *A*-values, *σ_f_*. For the vast majority of transitions, the values of *σ_A_* and *σ_f_* are very close to each other. For only 9 transitions out of total 229 listed in Table 3, *σ_A_* differs from *σ_f_* by more than 10%. Most of these transitions are very weak and have branching fractions smaller than 4%. The largest differences between *σ_A_* and *σ_f_* (by a factor of more than 3) occur for two transitions. One of them is the ^5^D_2_–^3^P2_2_ M1+E2 transition at 3,837.58 Å having a tiny branching fraction of 0.005% and an optimal *p* value of 0.096. The large difference of *σ_A_* from *σ_f_* for this transition is explained by the peculiarity of its statistical distribution. The normal probability plot for this transition looks very similar to the one shown in Figure 8a for another transition with a different optimal value of *p* = 2.23. Due to smallness of the branching fraction, peculiarity of this transition hardly matters in practice. However, another peculiar transition having *σ_A_* greater than *σ_f_* by a factor of 5 deserves a closer look. It is the ^3^P2_1_–^3^P2_2_ M1 transition at 66,872 Å. Its peculiarity is caused by the extremely large value of *p* = 2,621, which implies that the statistical distribution of trial data for this transition is extremely asymmetrical. Ninety five percent of trial *A*-values for this transition are distributed symmetrically around the nominal value of 4.5406 × 10^−2^ s^−1^ with a relative standard deviation of 0.013% (equal to *σ_f_*). However, the remaining 5% of trials produce much larger deviations, up to 3%, all on the negative side (*i.e.*, these trial *A*-values are smaller than the nominal value from the LSF). Since both *σ_A_* and *σ_f_* are very small for this transition (0.06% and 0.013%, respectively), this statistical peculiarity may not be of practical importance. However, one cannot rule out the presence of such peculiarities in other atomic problems, where they may have significant consequences.

The closeness of *σ_A_* and *σ_f_*, if it is confirmed for other types of transitions and for other atomic systems, may lead to a conclusion that investigation of shapes of statistical distributions is not really necessary. However, one should keep in mind that the standard deviations alone do not fully describe statistical properties of the calculated quantities. If derived from a sufficiently large statistical base, they do allow one to say that the “true” value of the calculated quantity is within the limits of plus or minus one standard deviation from the mean value with a probability of 68%. However, if one needs to know the limits of possible deviations more strictly, say, with a probability of 95%, the values of *σ* are of no use without additional knowledge of the shape of the distribution. Sufficient data about these shapes is provided in Table 3 by the values of *p* describing the optimal Box–Cox transformation. For the particular atomic problem considered here, most of them are clustered around 0.3 (see Figure 7). This means that statistical distributions of most considered transitions are skewed to the positive side, *i.e.*, there are greater number of trials with *A* > A* than with *A* < A*. Values of *p* > 1 result in negative skew, meaning that it is more probable to obtain *A* < A*. This information cannot be obtained from standard deviations alone. For the particular ^3^P2_1_–^3^P2_2_ M1 transition at 66,872 Å discussed above, knowledge of *σ_A_* = 0.06% allows one to say that the calculated *A*-value is (4.5406 ± 0.0027) × 10^−2^ s^−1^ at the 68% confidence level. However, if one needs error limits at the 95% confidence level, the result $(4.5406_{-0.0025}^{+0.0009})\times 10^{-2}\;s^{-1}$ can be obtained only if *p* and *σ_f_* are known.

As noted above, the standard deviations for straight *A* values are not of much use for statistical considerations. Nevertheless, they can be compared with similar estimates obtained earlier in [5] from comparisons with the calculations of Nahar *et al.* [6]. The latter authors used the Breit–Pauli R-matrix method to compute the E1, M1, and E2 transition probabilities of Fe V. The quality of their calculations was largely determined by the wavefunctions of the Fe VI ionic core calculated with the Breit–Pauli version of the SUPERSTRUCTURE code [9]. The accuracy of such calculations is usually similar to that of *ab initio* calculations with Cowan’s codes. The SUPERSTRUCTURE code, as well as Cowan’s codes, uses a non-relativistic approximation with relativistic corrections introduced as perturbations. The principal difference is that, to compute one-electron orbitals, SUPERSTRUCTURE uses a scaled statistical model of the Thomas–Fermi–Dirac potential, while Cowan’s HFR code uses hydrogenic orbitals. The R-matrix code then solves the (*e* + Fe VI) problem, which couples the bound states with the continuum and produces the calculated spectrum represented as continuum with multiple resonances. For bound-bound transitions, this introduces an additional problem of identification of the calculated resonances with initial and final bound states. Due to poor quality of wavefunctions (from SUPERSTRUCTURE), the energy levels calculated by Nahar *et al.* [6] differ from experimental ones by many thousands of reciprocal centimeters. For comparison, the standard deviation of calculated and experimental energy levels in my LSF with Cowan’s codes (described in Section 3.1) is only 117 cm^−1^. However, there are no other published calculations of M1 and E2 transition rates of Fe V, so the results of Nahar *et al.* [6] were compared in [5] with my Cowan-code calculations to estimate the uncertainties of the latter. Due to the low accuracy of the SUPERSTRUCTURE calculations, one would expect thus estimated uncertainties to be overly pessimistic. However, comparison with the standard deviations of *A*-values obtained here by the Monte Carlo method reveals that for a few transitions the uncertainties given in [5] were underestimated, and the corresponding category of accuracy should be degraded by one category (e.g., “C+” should be changed to “C”, and “B” to “C+”). These few transitions are marked with an asterisk in the *σ_A_* column. For the rest of the transitions, the uncertainties estimated in [5] are greater (in most cases much greater) than those found here by random trials. This confirms the general validity of the traditional method of critical assessment of uncertainties, but indicates that a small statistical basis of comparisons sometimes leads to underestimated uncertainties.

As noted in Section 3.3, for a few transitions the optimal Box–Cox transformation produces statistical distributions that are rather far from normal, meaning that there are too many trials in which transformed *A*-values deviate from the initial (LSF) ones by more than 5*σ*. Such abnormal transitions are marked in Table 3 with an asterisk in the last column.

### 3.5. Required Number of Comparisons

The number of comparisons required for a reliable estimation of uncertainty of calculated *A*-values can be estimated by comparing the values of *σ_A_* obtained with a progressively increasing number of trials. Such comparison shows that, with just ten random trials, the resulting *σ_A_* differs from the “true” value (obtained with 10,000 trials) by more than 20% for about 99% of all transitions. With 100 random trials, the number of such inaccurate determinations of *σ_A_* abruptly drops to about 3% of all transitions, and with 1,000 trials, it further decreases to about 1%. Thus, the number of calculations compared to each other, required for a reliable determination of uncertainties of *A*-values, is proportional to the number of transitions considered and should be about a million for 600 transitions considered here. With the maximum number of trials considered here, 10,000, one can expect to have a few transitions with an insufficiently accurate estimate of *σ_A_*. If the requirement on the accuracy of *σ_A_* is relaxed to 50% instead of 20% (let us call it “reasonable” instead of “accurate”), the needed number of comparisons drops drastically. Just ten trials then result in reasonable estimates of *σ_A_* for about 90% of all transitions, and with 100 trials there are only a few unreasonably bad estimates of *σ_A_*.

Of course, the above considerations apply only to the test case considered here, the M1 and E2 transitions of Fe V calculated with Cowan’s codes. Other codes and other atomic systems may have different properties, and it would be interesting to obtain similar estimates for them. This would lead to better understanding of dependability of traditional methods of uncertainty estimation based on comparison of results of different atomic codes.

### 3.6. Further Considerations

Statistical distributions of *A* values considered here were obtained using several arbitrary assumptions described in the Introduction. First of all, the variations of the input atomic parameters such as Slater and CI parameters were assumed to have normal statistical distributions. There is no physical or mathematical reason behind this assumption. A further investigation is needed in order to find actual shapes of these distributions and their influence on the final *A* values. The same is true for the E2 transition integrals. In addition, the widths of statistical distributions of the latter were arbitrarily assumed to be equal to a fixed value of 1%. Estimation of the actual variances of the E2 transition matrix elements requires a separate study. Furthermore, the various atomic parameters are strongly correlated. These correlations were accounted in the present work only partially, via linking some of the parameters in groups having fixed parameter ratios. The actual correlations are much more complex and should be investigated.

The method suggested here was relatively easy to implement with Cowan’s suite of codes, which include the LSF procedure with well-defined uncertainties of the fitted parameters. Other codes also have adjustable parameters. For example, SUPERSTRUCTURE [9] uses a set of adjustable scaling parameters ***λ*** of the Thomas–Fermi–Dirac potential. Some theorists adjust these parameters to produce energy levels most close to observed ones. This procedure is, in principle, similar to Cowan’s LSF. To make it a true LSF, one needs a matrix of derivatives [*∂****λ****/∂****E***] similar to [*∂****P****/∂****E***] in Equation (1). Then the uncertainties of adjusted ***λ*** could be estimated with a similar formula. I am not aware if such procedure is implemented in SUPERSTRUCTURE, and I never saw uncertainties of ***λ*** reported in publications. However, even if the adjustment of ***λ*** is manual, one can obtain some idea of their uncertainties by trial and error, and the resulting uncertainties in *A*-values can be estimated by a method similar to the one described here.

Adjustable parameters are also used by Hibbert in the CIV3 code [10]. Namely, in the process that he calls “fine tuning”, he adjusts the diagonal Hamiltonian matrix elements to achieve the best agreement of calculated energies with experiment [11]. This adjustment is not much different from adjusting *E*_av_ in Cowan’s LSF. Thus, it must be possible to investigate the uncertainty of this fine tuning and its effect on *A*-values.

Other sophisticated *ab initio* atomic codes implementing multi-configuration Hartree–Fock and Dirac–Fock methods [12,13] also compute the Slater and CI parameters. However, in the current implementation of these codes these parameters cannot be adjusted, which is quite unfortunate. To implement the described method with these codes, they must be modified. Estimation of uncertainties of *ab initio* calculations without the use of experimental data would require different methods. However, these methods must necessarily involve an analysis of error propagation through matrix equations.

In principle, a clever statistician should be able to predict the expected transformations of distribution functions of calculated *A*-values on the basis of known matrix-diagonalization procedures and formulas for summation of various contributions to the line strength used in atomic codes, avoiding the computationally expensive empirical method used here. So far, I could not find any methods for that. In-depth investigation of statistical properties of atomic calculations requires much more than basic training in applied statistics. The present work shows only a first glimpse of these properties and gives more questions than answers. Some aspects of the problems touched upon appear to be on the cutting edge of statistical theory.

Solving these problems calls for development of a relatively new field of atomic physics, *statistical atomic physics*, the main subjects of which should be investigation of statistical properties of atomic parameters and propagation of errors through atomic and plasma-kinetic models. Some studies related to this field have already been published. They involve such topics as unresolved transition arrays, chaos theories for spectra of complex atomic systems with open *f*-shell, and Monte Carlo simulations of plasma kinetics. However, in view of the present findings, statistical considerations used in these studies seem to be rather naïve. Proper treatment of statistical distributions and correlations of atomic properties requires a deep knowledge of both atomic physics and applied statistics. Thus, a new generation of specialists possessing the needed skills should be created.

## 4. Conclusions

The present work suggests a method for evaluation of sensitivity of calculated transition probabilities (*A* values) to small variations of input atomic parameters by Monte Carlo random trials. This method provides an insight into shapes of statistical distributions of the *A* values. These distributions were found to be far from normal for most M1 and E2 transitions within the ground configuration of Fe V, which were used as a test problem. However, for almost all transitions, it turned out possible to find an optimal Box–Cox transformation that results in near normal statistical distribution. If the range of possible random variations of the input parameters is known, e.g., from a least-squares fitting of Slater parameters, this method leads to statistically sound estimates of internal uncertainties of the particular computational atomic model used.

In order to determine the total uncertainties of the calculation, these internal uncertainty estimates should be combined with studies of effects of approximations made in the atomic model, for example, by comparing *A* values obtained with increasing number of included basis states, estimating convergence trends, and extrapolating these trends to an infinite basis size. Additionally, the calculated *A* values should be compared with other independent ones based on different atomic models of comparable quality (for example, non-relativistic calculations with perturbative account for relativistic effects can be compared with fully relativistic calculations). Neither of these additional uncertainty estimates was made in the present work, so the numerical results given here represent the lower bounds of uncertainties. Even so, they helped detect and correct uncertainties underestimated earlier by other methods.

One conclusion following from the present results is that uncertainties should be estimated not for the straight *A* values, but for the transformed ones that have a statistical distribution close to normal. This requires that, for each transition, the optimized transformation parameter *p* should be given along with the *A* value and an estimate of *σ* for the transformed *A* value.

When results of two different theoretical models are compared to each other, one can expect that the shapes of statistical distributions of the *A* value for the same transition should be similar for different calculations, at least if cancellation is weak. This statement is not substantiated by any factual data, but is based on a general consideration of the method of computation of line strengths in all currently available atomic codes. Expanding the basis set should only add some minor terms in the summation series of these calculations. Thus, a significant change in the distribution shape should be expected only for transitions with strong cancellation.

There is an important implication for Monte Carlo simulations of plasma kinetics, namely, the distributions of the input atomic parameters (such as the *A* values and collision rates) should not be assumed normal. Instead, they must be skewed according to a Box–Cox transformation optimized individually for each transition.

## Figures and Tables

**Table 1:** Parameters of the least-squares fitting (LSF) used as input for Monte Carlo trials (in units of cm^−1^).

Configurations		Parameter	LSF	*σ*	Group a	HFR	LSF/HFR
3d^4^		*E*_av_	36,510.2	43		0.0	
		*F*^2^(3d3d)	90,868.6	118		105,204.6	0.8637
		*F*^4^(3d3d)	55,549.7	191		66,193.4	0.8392
		*α*_3d_	36.8	3		0.0	
		*β*_3d_	599.6	61		0.0	
		*T*_3d_	−7.7	0	14	0.0	
		*ς*_3d_	531.9	23		533.1	0.9977
3d^3^4s		*E*_av_	215,050.4	31		177,245.6	1.2133
		*F*^2^(3d3d)	95,607.2	135		111,187.6	0.8599
		*F*^4^(3d3d)	58,863.0	209		70,214.5	0.8383
		*α*_3d_	45.2	3		0.0	
		*β*_3d_	634.1	60		0.0	
		*T*_3d_	−7.7	0	14	0.0	
		*ς*_3d_	586.8	24		585.2	1.0027
		*G*^2^(3d4s)	10,704.7	75	6	12,235.1	0.8749
3d^3^5s		*E*_av_	421,078.0	58	5	382,121.1	1.1019
		*F*^2^(3d3d)	97,235.2	815		112,221.2	0.8665
		*F*^4^(3d3d)	59,610.7	1157		70,920.1	0.8405
		*α*_3d_	52.9	7		0.0	
		*β*_3d_	362.6	362		0.0	
		*T*_3d_	−7.7	0	14	0.0	
		*ς*_3d_	568.4	31		592.4	0.9595
		*G*^2^(3d5s)	3327.7	101		3315.5	1.0037
3d^3^4d		*E*_av_	387,478.0	30		348,713.5	1.1112
		*F*^2^(3d3d)	96,771.8	95	1	112,024.1	0.8638
		*F*^4^(3d3d)	59,294.2	174	2	70,789.8	0.8376
		*α*_3d_	45.5	3	12	0.0	
		*β*_3d_	495.8	51	13	0.0	
		*T*_3d_	−7.7	0	14	0.0	
		*ς*_3d_	589.0	16	3	590.3	0.9978
		*ς*_4d_	75.8	fixed		76.5	0.9908
		*F*^2^(3d4d)	16,966.1	126	7	19,123.3	0.8872
		*F*^4^(3d4d)	8,158.7	177	8	8,715.8	0.9361
		*G*°(3d4d)	5,223.2	28	9	8,263.5	0.6321
		*G*^2^(3d4d)	6,488.4	103	10	7,994.5	0.8116
		*G*^4^(3d4d)	5,643.9	132	11	5,877.2	0.9603
3d^3^5d		*E*_av_	486,707.5	67	5	450,809.1	1.0796
		*F*^2^(3d3d)	97,062.4	96	1	112,360.5	0.8638
		*F*^4^(3d3d)	59,483.7	174	2	71,016.1	0.8376
		*α*_3d_	45.5	3	12	0.0	
		*β*_3d_	495.8	51	13	0.0	
		*T*_3d_	−7.7	0	14	0.0	
		*ς*_3d_	592.0	17	3	593.3	0.9978
		*ς*_5d_	33.9	fixed		34.2	
		*F*^2^(3d5d)	6,810.0	51	7	7,675.8	0.8872
		*F*^4^(3d5d)	3,290.4	71	8	3,515.1	0.9361
		*G*°(3d5d)	1,960.2	10	9	3,101.2	0.6321
		*G*^2^(3d5d)	2,624.3	42	10	3,233.5	0.8116
		*G*^4^(3d5d)	2,337.2	55	11	2,433.8	0.9603
3d^2^4s^2^		*E*_av_	447,291.0	62	5	411,400.0	1.0872
		*F*^2^(3d3d)	101,108.2	100	1	117,043.9	0.8638
		*F*^4^(3d3d)	62,117.5	182	2	74,160.5	0.8376
		*α*_3d_	45.5	3	12	0.0	
		*β*_3d_	495.8	51	13	0.0	
		*T*_3d_	−7.7	0	14	0.0	
		*ς*_3d_	639.4	18	3	640.8	0.9978
3d^2^4s4d		*E*_av_	623,244.0	86	5	587,320.0	1.0612
		*F*^2^(3d3d)	101,728.1	100	1	117,761.5	0.8638
		*F*^4^(3d3d)	62,532.2	183	2	74,655.6	0.8376
		*α*_3d_	45.5	3	12	0.0	
		*β*_3d_	495.8	51	13	0.0	
		*T*_3d_	−7.7	0	14	0.0	
		*ς*_3d_	644.1	18	3	645.5	0.9978
		*ς*_4d_	85.4	fixed		86.2	0.9907
		*F*^2^(3d4d)	18,159.0	135	7	20,467.8	0.8872
		*F*^4^(3d4d)	8,755.3	189	8	9,353.2	0.9361
		*G*^2^(3d4s)	10,739.7	75	6	12,275.2	0.8749
		*G*°(3d4d)	5,514.6	29	9	8,724.6	0.6321
		*G*^2^(3d4d)	6,889.7	110	10	8,488.9	0.8116
		*G*^4^(3d4d)	6,021.5	141	11	6,270.5	0.9603
		*G*^2^(4s4d)	30,469.1	fixed		33,996.9	0.8962
3d^2^4d^2^		*E*_av_	811,506.1	112	5	775,546.8	1.0464
		*F*^2^(3d3d)	102,346.9	101	1	118,477.8	0.8638
		*F*^4^(3d3d)	62,946.2	185	2	75,149.9	0.8376
		*α*_3d_	45.5	3	12	0.0	
		*β*_3d_	495.8	51	13	0.0	
		*T*_3d_	−7.7	0	14	0.0	
		*F*^2^(4d4d)	33,810.3	fixed		39,233.4	0.8618
		*F*^4^(4d4d)	23,909.6	fixed		26,600.9	0.8988
		*ς*_3d_	649.0	18	3	650.4	0.9978
		*ς*_4d_	90.6	fixed		91.4	0.9912
		*F*^2^(3d4d)	18,870.5	141	7	21,269.8	0.8872
		*F*^4^(3d4d)	9,140.4	198	8	9,764.6	0.9361
		*G*°(3d4d)	5,687.7	30	9	8,998.4	0.6321
		*G*^2^(3d4d)	7,150.7	114	10	8,810.5	0.8116
		*G*^4^(3d4d)	6,265.5	147	11	6,524.5	0.9603

Configuration interaction							

3d^4^	−3d^3^4s	*R*^2^(3d3d, 3d4s)	2,217.4	95	15	3,336.6	0.6646
	−3d^3^5s	*R*^2^(3d3d, 3d5s)	1,219.4	52	15	1,834.8	0.6646
	−3d^3^4d	*R*°(3d3d, 3d4d)	1,821.3	78	15	2,740.5	0.6646
		*R*^2^(3d3d, 3d4d)	12,948.9	557	15	19,484.5	0.6646
		*R*^4^(3d3d, 3d4d)	8,883.0	382	15	13,366.4	0.6646
	−3d^3^5d	*R*°(3d3d, 3d5d)	1,047.9	45	15	1,576.8	0.6646
		*R*^2^(3d3d, 3d5d)	7,329.0	315	15	11,028.1	0.6646
		*R*^4^(3d3d, 3d5d)	5,046.3	217	15	7,593.2	0.6646
	−3d^2^4s^2^	*R*^2^(3d3d, 3s3s)	10,004.3	430	15	15,053.6	0.6646
	−3d^2^4s4d	*R*^2^(3d3d, 4s4d)	6,419.5	276	15	9,659.5	0.6646
	−3d^2^4d^2^	*R*°(3d3d, 4d4d)	4,952.9	213	15	7,452.7	0.6646
		*R*^2^(3d3d, 4d4d)	6,174.4	266	15	9,290.7	0.6646
		*R*^4^(3d3d, 4d4d)	4,676.0	201	15	7,036.0	0.6646
3d^3^4s	−3d^3^5s	*R*°(3d4s, 3d5s)	409.1	18	15	615.6	0.6645
		*R*^2^(3d4s, 5s3d)	4,080.3	175	15	6,139.6	0.6646
	−3d^3^4d	*R*^2^(3d4s, 3d4d)	14,076.1	605	15	21,180.5	0.6646
		*R*^2^(3d4s, 4d3d)	5,091.7	219	15	7,661.5	0.6646
	−3d^3^5d	*R*^2^(3d4s, 3d5d)	8,682.3	373	15	13,064.4	0.6646
		*R*^2^(3d4s, 5d3d)	3,471.1	149	15	5,223.1	0.6646
	−3d^2^4s^2^	*R*^2^(3d3d, 3d3s)	3,873.0	167	15	5,827.8	0.6646
	−3d^2^4s4d	*R*°(3d3d, 3d4d)	1,926.3	83	15	2,898.5	0.6646
		*R*^2^(3d3d, 3d4d)	13,783.0	593	15	20,739.5	0.6646
		*R*^4^(3d3d, 3d4d)	9,476.2	408	15	14,258.9	0.6646
		*R*^2^(3d4s, 4s4d)	−8,252.2	355	15	−12,417.2	0.6646
		*R*°(3d4s, 4d4s)	−796.0	34	15	−1,197.8	0.6646
	−3d^2^4d^2^	*R*^2^(3d4s, 4d4d)	−3,905.8	168	15	−5,877.1	0.6646
3d^3^5s	−3d^3^4d	*R*^2^(3d5s, 3d4d)	3,396.2	146	15	5,110.4	0.6646
		*R*^2^(3d5s, 4d3d)	2,475.6	106	15	3,725.1	0.6646
	−3d^3^5d	*R*^2^(3d5s, 3d5d)	4,378.2	188	15	6,588.0	0.6646
		*R*^2^(3d5s, 5d3d)	1,743.1	75	15	2,622.8	0.6646
	−3d^2^4s4d	*R*^2^(3d5s, 4s4d)	185.6	8	15	279.3	0.6645
		*R*°(3d5s, 4d4s)	2,099.6	90	15	3,159.3	0.6646
	−3d^2^4d^2^	*R*^2^(3d5s, 4d4d)	954.7	41	15	1,436.6	0.6646
3d^3^4d	−3d^3^5d	*R*°(3d4d, 3d5d)	501.5	22	15	754.6	0.6646
		*R*^2^(3d4d, 3d5d)	6,530.5	281	15	9,826.6	0.6646
		*R*^4^(3d4d, 3d5d)	3,481.2	150	15	5,238.2	0.6646
		*R*°(3d4d, 5d3d)	3,347.1	144	15	5,036.5	0.6646
		*R*^2^(3d4d, 5d3d)	3,346.7	144	15	5,035.8	0.6646
		*R*^4^(3d4d, 5d3d)	2,486.7	107	15	3,741.8	0.6646
	−3d^2^4s^2^	*R*^2^(3d4d, 3s3s)	−7,061.3	304	15	−10,625.3	0.6646
	−3d^2^4s4d	*R*^2^(3d3d, 3d4s)	4,209.3	181	15	6,333.8	0.6646
		*R*^2^(3d4d, 4s4d)	−6,388.1	275	15	−9,612.2	0.6646
		*R*^2^(3d4d, 4d4s)	−2,959.1	127	15	−4,452.6	0.6646
	−3d^2^4d^2^	*R*°(3d3d, 3d4d)	1,955.1	84	15	2,941.9	0.6646
		*R*^2^(3d3d, 3d4d)	13,992.6	602	15	21,054.9	0.6646
		*R*^4^(3d3d, 3d4d)	9,625.4	414	15	14,483.5	0.6646
		*R*°(3d4d, 4d4d)	80.7	3	15	121.4	0.6647
		*R*^2^(3d4d, 4d4d)	−2,931.1	126	15	−4,410.5	0.6646
		*R*^4^(3d4d, 4d4d)	−2,127.2	91	15	−3,200.8	0.6646
3d^3^5d	−3d^2^4s^2^	*R*^2^(3d5d, 3s3s)	−3,819.9	164	15	−5,747.9	0.6646
	−3d^2^4s4d	*R*^2^(3d5d, 4s4d)	−2,351.0	101	15	−3,537.6	0.6646
		*R*^2^(3d5d, 4d4s)	−1,316.3	57	15	−1,980.6	0.6646
	−3d^2^4d^2^	*R*°(3d5d, 4d4d)	1,550.6	67	15	2,333.2	0.6646
		*R*^2^(3d5d, 4d4d)	−657.1	28	15	−988.7	0.6646
		*R*^4^(3d5d, 4d4d)	−769.7	33	15	−1,158.1	0.6646
3d^2^4s^2^	−3d^2^4s4d	*R*^2^(3d3s, 3d4d)	15,121.7	650	15	22,753.9	0.6646
		*R*^2^(3d3s, 4d3d)	5,433.2	234	15	8,175.4	0.6646
	−3d^2^4d^2^	*R*^2^(3s3s, 4d4d)	23,912.3	1,028	15	35,981.2	0.6646
3d^2^4s4d	−3d^2^4d^2^	*R*^2^(3d4s, 3d4d)	15,680.1	674	15	23,594.1	0.6646
		*R*^2^(3d4s, 4d3d)	5,650.7	243	15	8,502.7	0.6646
		*R*^2^(4s4d, 4d4d)	23,674.3	1,018	15	35,623.1	0.6646

E2 transition reduced matrix elements							

3d^4^	−3d^4^	(3d, 3d)				−1.22129	
3d^4^	−3d^3^4s	(3d, 4s)				−36.36873	
3d^4^	−3d^3^5s	(3d, 5s)				−9.56604	
3d^4^	−3d^3^4d	(3d, 4d)				1.04638	
3d^4^	−3d^3^5d	(3d, 5d)				0.40754	
3d^3^4s	−3d^4^	(4s, 3d)				−1.22729	
3d^3^4s	−3d^3^4s	(3d, 3d)				−1.10293	
3d^3^4s	−3d^3^4d	(4s, 4d)				5.48000	
3d^3^4s	−3d^3^5d	(4s, 5d)				0.09788	
3d^3^4s	−3d^2^4s^2^	(3d, 4s)				−1.05636	
3d^3^4s	−3d^2^4s4d	(3d, 4d)				0.90280	
3d^3^5s	−3d^4^	(5s, 3d)				−0.09184	
3d^3^5s	−3d^3^5s	(3d, 3d)				−1.08225	
3d^3^5s	−3d^3^4d	(5s, 4d)				−9.51515	
3d^3^5s	−3d^3^5d	(5s, 5d)				17.67485	
3d^3^4d	−3d^4^	(4d, 3d)				1.04638	
3d^3^4d	−3d^3^4s	(4d, 4s)				5.48000	
3d^3^4d	−3d^3^5s	(4d, 5s)				−9.51515	
3d^3^4d	−3d^3^4d	(4d, 4d)				−10.89202	
3d^3^4d	−3d^3^5d	(4d, 5d)				6.90270	
3d^3^4d	−3d^2^4s4d	(3d, 4s)				−1.02367	
3d^3^4d	−3d^2^4d^2^	(3d, 4d)				0.87600	
3d^3^5d	−3d^4^	(5d, 3d)				0.40754	
3d^3^5d	−3d^3^4s	(5d, 4s)				0.09788	
3d^3^5d	−3d^3^5s	(5d, 5s)				17.67485	
3d^3^5d	−3d^3^4d	(5d, 4d)				6.90270	
3d^3^5d	−3d^3^5d	(5d, 5d)				−36.43838	
3d^2^4s^2^	−3d^3^4s	(4s, 3d)				−1.05636	
3d^2^4s^2^	−3d^2^4s^2^	(3d, 3d)				−1.00354	
3d^2^4s^2^	−3d^2^4s4d	(4s, 4d)				5.14550	
3d^2^4s4d	−3d^3^4s	(4d, 3d)				0.90280	
3d^2^4s4d	−3d^3^4d	(4s, 3d)				−1.02367	
3d^2^4s4d	−3d^2^4s^2^	(4d, 4s)				5.14550	
3d^2^4s4d	−3d^2^4s4d	(4d, 4d)				−9.84869	
3d^2^4s4d	−3d^2^4d^2^	(4s, 4d)				4.98748	
3d^2^4d^2^	−3d^3^4d	(4d, 3d)				0.87600	
3d^2^4d^2^	−3d^2^4s4d	(4d, 4s)				4.98748	
3d^2^4d^2^	−3d^2^4d^2^	(4d, 4d)				−9.45614	

a Parameters in each numbered group were linked together with their ratio fixed at the Hartree–Fock level.

**Table 2:** Fractional number of outliers deviating from the mean by more than *n* standard deviations for the normal statistical distribution, *N*_norm_(*n*).

*n*	*N_norm_*(n)
−5	0.000000286652
−4	0.000031671242
−3	0.001349898032
−2	0.022750131948
−1	0.158655253932
−0	0.500000000000
+0	0.500000000000
+1	0.158655253932
+2	0.022750131948
+3	0.001349898032
+4	0.000031671242
+5	0.000000286652

**Table 3:** Calculated transition probabilities of M1 and E2 transitions of Fe V with estimated uncertainties and Box–Cox transformation parameters optimized for each transition, based on overall statistics for five sets of Monte Carlo trials, four with 1,000 trials each and one with 10,000 trials.

Transition	*E*_low_, cm^− 1^	*E*_up_ cm^−1^	*λ*_Ritz_,^a^ Å	*A*, s^−1^	*σ_A_*,^b^ %	CF c	BF d	Type e	Fraction(E2)^f^	*p* g	*σ_f_*,^i^ %
^3^P2_1_–^1^S1_0_	24,972.8	121,130.1	1,039.963	6.3 × 10^−1^	9	0.0440(3)	0.0073(6)	M1	0	0.484(21)	9
^3^F2_2_–^1^S1_0_	26,760.7	121,130.1	1,059.666	2.54 × 10^−1^	9	0.0625(7)	0.00294(22)	E2	1	0.74(6)	9
^3^D_2_–^1^S1_0_	36,758.2	121,130.1	1,185.229	8.1 × 10^−1^	9	0.1150(13)	0.0094(8)	E2	1	0.40(4)	9
^1^D2_2_–^1^S1_0_	46,291.1	121,130.1	1,336.20	75.7	3	−0.1904(19)	0.878(5)	E2	1	0.88(21)	3
^3^P2_0_–^1^D1_2_	24,055.5	93,832.5	1,433.137	3.3 × 10^−2^	8	0.0229(5)	0.00083(6)	E2	1	0.55(4)	8
^3^H_4_–^1^D1_2_	24,932.4	93,832.5	1,451.377	2.10 × 10^−1^	9	0.1449(8)	0.0052(4)	E2	1	0.42(3)	9
^3^P2_2_–^1^D1_2_	26,468.2	93,832.5	1,484.466	6.6 × 10^−2^	9	0.00398(5)	0.00165(14)	M1 + E2	0.092(3)	0.12(3)	9
^3^F2_4_–^1^D1_2_	26,973.7	93,832.5	1,495.689	2.89 × 10 ^−1^	6	−0.1997(12)	0.0072(4)	E2	1	0.91(5)	6
^3^G_4_–^1^D1_2_	30,147.2	93,832.5	1,570.221	3.1 × 10^−1^	12	0.2507(8)	0.0076(9)	E2	1	0.347(19)	12
^5^D_1_–^3^P1_0_	142.4	63,419.8	1,580.343	8.6 × 10^−1^	8	0.01802(15)	0.193(13)	M1	0	0.527(23)	8
^5^D_2_–^3^P1_0_	417.5	63,419.8	1,587.244	4.5 × 10^−2^	8	0.1840(6)	0.0101(7)	E2	1	0.48(3)	8
^5^D_0_–^3^P1_1_	0.0	62,914.1	1,589.47	9.4 × 10^−2^	8	0.00491(5)	0.0212(13)	M1	0	0.539(23)	8
^5^D_1_–^3^P1_1_	142.4	62,914.1	1,593.075	9.4 × 10^−3^	9	0.139(3)	0.00213(14)	M1 + E2	0.9781(18)	0.45(3)	9
^5^D_2_–^3^P1_1_	417.5	62,914.1	1,600.087	6.9 × 10^−1^	8	0.0634(4)	0.156(10)	M1 + E2	0.0191(4)	0.505(22)	8
^5^D_1_–^3^F1_2_	142.4	62,321.1	1,608.268	2.15 × 10^−2^	8	0.70(3)	0.00290(19)	M1 + E2	0.0398(13)	0.61(3)	8
^5^D_3_–^3^P1_1_	803.1	62,914.1	1,610.021	7.6 × 10^−3^	9	0.0853(4)	0.00171(11)	E2	1	0.43(3)	9
^5^D_2_–^3^F1_3_	417.5	62,364.3	1,614.288	3.9 × 10^−2^	8	0.0372(4)	0.0057(4)	M1	0.000396(21)	0.58(3)	8
^5^D_2_–^3^F1_2_	417.5	62,321.1	1,615.415	4.6 × 10^−2^	8	0.0164(4)	0.0062(4)	M1 + E2	0.0128(4)	0.526(24)	8
^5^D_0_–^3^P1_2_	0.0	61,854.1	1,616.71	4.0 × 10^−3^	10	0.112(3)	0.00112(8)	E2	1	0.39(3)	10
^5^D_1_–^3^P1_2_	142.4	61,854.1	1,620.438	3.8 × 10^−2^	9	0.00943(20)	0.0108(7)	M1 + E2	0.101(3)	0.492(21)	9
^5^D_3_–^3^F1_3_	803.1	62,364.3	1,624.400	1.21 × 10^−1^	9	0.00286(3)	0.0176(14)	M1 + E2	0.0132(4)	0.496(23)	9
^5^D_3_–^3^F1_2_	803.1	62,321.1	1,625.540	1.62 × 10^−2^	9	0.0152(4)	0.00219(18)	M1 + E2	0.1097(23)	0.407(17)	9
^5^D_3_–^3^F1_4_	803.1	62,238.0	1,627.739	4.0 × 10^−2^	8	0.01356(19)	0.0059(4)	M1 + E2	0.0379(8)	0.548(25)	8
^5^D_4_–^3^F1_3_	1,282.7	62,364.3	1,637.154	2.35 × 10^−2^	9	0.0330(5)	0.0034(3)	M1 + E2	0.194(4)	0.478(23)	9
^5^D_3_–^3^P1_2_	803.1	61,854.1	1,637.975	4.9 × 10^−1^	9	0.919(6)	0.138(9)	M1	0.00893(19)	0.470(22)	9
^5^D_4_–^3^F1_4_	1,282.7	62,238.0	1,640.546	2.39 × 10^−1^	9	0.00509(10)	0.035(3)	M1 + E2	0.0405(9)	0.450(22)	9
^3^P1_1_–^1^S1_0_	62,914.1	121,130.1	1,717.74	3.6	9	−0.9999951(4)	0.042(4)	M1	0	0.448(18)	9
^1^G2_4_–^1^D1_2_	36,585.6	93,832.5	1,746.819	26.5	2.0	0.3386(15)	0.660(4)	E2	1	0.29(13)	2.0
^3^D_3_–^1^D1_2_	36,630.0	93,832.5	1,748.175	5.5 × 10^−1^	9	−0.883(5)	0.0138(11)	M1	0.00534(17)	0.484(20)	9
^3^D_2_–^1^D1_2_	36,758.2	93,832.5	1,752.102	1.85 × 10^−1^	9	0.0826(14)	0.0046(4)	M1 + E2	0.407(6)	0.470(22)	9
^3^D_1_–^1^D1_2_	36,925.2	93,832.5	1,757.244	5.6 × 10^−1^	9	−0.9550(18)	0.0139(12)	M1	0.000143(12)	0.451(19)	9
^1^S2_0_–^1^D1_2_	39,633.0	93,832.5	1,845.04	1.94	2.5	0.0641(10)	0.0482(7)	E2	1	0.09(10)	2.5
^5^D_4_–^1^F_3_	1,282.7	52,732.6	1,943.64	9.0 × 10^−4^	18	0.0320(7)	0.00138(12)	M1 + E2	0.0138(4)	0.237(10)	18
^1^D2_2_–^1^D1_2_	46,291.1	93,832.5	2,102.76	6.76	1.9	0.3210(14)	0.1682(10)	E2	0.99953(9)	0.66(16)	1.9
^3^H_4_–^1^G1_4_	24,932.4	71,280.3	2,156.92	1.08 × 10^−1^	6	0.00095(7)	0.0228(9)	M1 + E2	0.0205(19)	0.98(3)	6
^3^H_5_–^1^G1_4_	25,225.5	71,280.3	2,170.65	2.9 × 10^−1^	10	1.0000000(0)	0.062(4)	M1	0.00286(11)	0.398(19)	10
^5^D_2_–^1^D2_2_	417.5	46,291.1	2,179.22	2.0 × 10^−3^	18	−0.003989(14)	0.00156(14)	M1	0.00082(4)	0.242(10)	17
^3^F2_3_–^1^G1_4_	26,842.3	71,280.3	2,249.63	2.28 × 10^−1^	10	−0.0598(12)	0.048(4)	M1	0.00560(16)	0.380(19)	10
^3^F2_4_–^1^G1_4_	26,973.7	71,280.3	2,256.30	2.8 × 10^−1^	9	−0.000310(9)	0.058(4)	M1	0.00086(16)	0.431(22)	9
^3^G_3_–^1^G1_4_	29,817.1	71,280.3	2,411.04	2.30 × 10^−1^	7	−0.274(14)	0.0485(21)	M1	0.00355(8)	0.76(3)	7
^1^F_3_–^1^D1_2_	52,732.6	93,832.5	2,432.36	1.79	3	0.833(6)	0.0445(8)	E2	0.9980(4)	1.00(21)	3
^3^G_5_–^1^G1_4_	30,429.9	71,280.3	2,447.22	2.81 × 10^−1^	8	0.716(14)	0.059(3)	M1 + E2	0.0242(6)	0.537(19)	8
^3^P2_0_–^3^P1_1_	24,055.5	62,914.1	2,572.66	1.72 × 10^−2^	11	−0.000017(2)	0.0039(4)	M1	0	0.343(22)	11
^3^P2_1_–^3^P1_0_	24,972.8	63,419.8	2,600.21	6.3 × 10^−3^	6	0.0000020(1)	0.00142(9)	M1	0	0.32(6)	6
^3^P2_0_–^3^F1_2_	24,055.5	62,321.1	2,612.53	1.98 × 10^−1^	2.2	0.1249(15)	0.0267(6)	E2	1	0.75(13)	2.2
^3^P2_1_–^3^P1_1_	24,972.8	62,914.1	2,634.86	3.23 × 10^−1^	2.3	0.221(7)	0.073(3)	E2	0.9976(4)	0.03(15)	2.3
^3^P2_0_–^3^P1_2_	24,055.5	61,854.1	2,644.81	3.07 × 10^−1^	2.1	0.295(5)	0.087(3)	E2	1	−0.06(17)	2.1
^3^H_4_–^3^F1_3_	24,932.4	62,364.3	2,670.72	5.34 × 10^−1^	3	0.749(16)	0.0778(10)	E2	0.99917(18)	0.00(15)	3
^3^P2_1_–^3^F1_3_	24,972.8	62,364.3	2,673.61	2.59 × 10^−1^	2.1	0.1276(11)	0.0377(6)	E2	1	0.75(12)	2.1
^3^H_4_–^3^F1_2_	24,932.4	62,321.1	2,673.81	2.69	2.1	0.763(10)	0.363(8)	E2	1	0.62(22)	2.1
^3^P2_1_–^3^F1_2_	24,972.8	62,321.1	2,676.70	2.15 × 10^−1^	2.2	0.1443(11)	0.0290(4)	E2	0.99926(12)	0.49(19)	2.2
^3^H_4_–^3^F1_4_	24,932.4	62,238.0	2,679.77	8.5 × 10^−3^	4	0.053(17)	0.00125(6)	M1 + E2	0.83(4)	−0.04(3)	4
^3^H_5_–^3^F1_3_	25,225.5	62,364.3	2,691.80	2.40	2.0	0.804(6)	0.350(6)	E2	1	0.56(18)	2.0
^3^H_5_–^3^F1_4_	25,225.5	62,238.0	2,700.99	4.05 × 10^−1^	3	0.831(7)	0.0596(4)	E2	0.9975(3)	0.15(15)	3
^3^P2_2_–^3^P1_0_	26,468.2	63,419.8	2,705.44	1.90	2.2	0.349(7)	0.425(9)	E2	1	0.20(4)	2.2 *
^5^D_0_–^3^D_1_	0.0	36,925.2	2,707.37	2.54 × 10^−1^	9	−0.647(11)	0.3478(13)	M1	0	0.508(20)	9
^3^P2_1_–^3^P1_2_	24,972.8	61,854.1	2,710.60	6.72 × 10^−1^	2.1	0.301(6)	0.190(4)	M1 + E2	0.963(4)	0.04(17)	2.1
^5^D_1_–^3^D_1_	142.4	36,925.2	2,717.86	2.24 × 10^−1^	8	−0.153(3)	0.3072(17)	M1	0.00242(7)	0.561(21)	8
^3^H_6_–^3^F1_4_	25,528.4	62,238.0	2,723.28	2.58	1.9	0.9692(14)	0.380(6)	E2	1	0.41(16)	1.9
^3^F2_2_–^3^P1_0_	26,760.7	63,419.8	2,727.03	4.04 × 10^−1^	4	0.067(3)	0.090(4)	E2	1	7.05(9)	3
^5^D_1_–^3^D_2_	142.4	36,758.2	2,730.25	2.12 × 10^−1^	9	−0.1428(8)	0.3656(9)	M1	8.2(6) × 10^−6^	0.479(20)	9
^5^D_2_–^3^D_1_	417.5	36,925.2	2,738.34	3.3 × 10^−3^	17	0.00369(17)	0.0045(4)	M1 + E2	0.045(4)	0.252(11)	16
^3^P2_2_–^3^P1_1_	26,468.2	62,914.1	2,742.98	1.49	2.2	0.413(11)	0.336(10)	M1 + E2	0.9865(12)	2.35(22)	2.2 *
^5^D_2_–^3^D_2_	417.5	36,758.2	2,750.92	1.70 × 10^−1^	8	−0.01167(15)	0.294(3)	M1	0.00299(7)	0.572(21)	8
^5^D_2_–^3^D_3_	417.5	36,630.0	2,760.66	1.06 × 10^−1^	9	−0.0764(3)	0.1526(4)	M1	7(22) × 10^−8^	0.444(19)	9
^3^F2_2_–^3^P1_1_	26,760.7	62,914.1	2,765.17	1.38 × 10^−1^	6	0.0713(22)	0.0312(13)	E2	0.9953(9)	−6.08(5)	5 *
^3^F2_3_–^3^P1_1_	26,842.3	62,914.1	2,771.43	2.55 × 10^−1^	2.5	0.0737(9)	0.0577(17)	E2	1	0.82(8)	2.5
^5^D_3_–^3^D_2_	803.1	36,758.2	2,780.43	1.14 × 10^−1^	11	−0.0543(7)	0.198(3)	M1	0.000132(6)	0.332(17)	11
^3^P2_2_–^3^F1_3_	26,468.2	62,364.3	2,785.00	1.27 × 10^−1^	3	0.132(6)	0.0185(5)	E2	0.9997(9)	8.19(18)	2.4
^3^P2_2_–^3^F1_2_	26,468.2	62,321.1	2,788.35	3.3 × 10^−2^	10	0.122(3)	0.0045(4)	M1 + E2	0.9793(25)	−4.53(4)	7
^5^D_3_–^3^D_3_	803.1	36,630.0	2,790.38	9.8 × 10^−2^	8	−0.003092(15)	0.1408(6)	M1	0.00552(11)	0.513(20)	8
^5^D_3_–^1^G2_4_	803.1	36,585.6	2,793.84	1.08 × 10^−3^	18	−0.03628(17)	0.00110(11)	M1	0.00299(6)	0.238(10)	18
^3^P2_2_–^3^F1_4_	26,468.2	62,238.0	2,794.83	3.32 × 10^−1^	2.1	0.1395(10)	0.0488(9)	E2	1	0.52(11)	2.1
^3^F2_2_–^3^F1_3_	26,760.7	62,364.3	2,807.88	3.44 × 10^−1^	3	0.196(9)	0.0501(9)	M1 + E2	0.744(15)	−3.21(20)	3 *
^3^F2_2_–^3^F1_2_	26,760.7	62,321.1	2,811.29	8.01 × 10^−1^	2.1	0.271(5)	0.1081(22)	E2	0.99969(5)	0.49(13)	2.1
^3^F2_3_–^3^F1_3_	26,842.3	62,364.3	2,814.33	4.77 × 10^−1^	3	0.170(8)	0.0695(20)	M1 + E2	0.979(4)	−0.02(15)	3
^3^F2_3_–^3^F1_2_	26,842.3	62,321.1	2,817.76	9.7 × 10^−1^	3	0.3846(24)	0.1305(14)	M1 + E2	0.929(3)	0.41(14)	3
^3^F2_2_–^3^F1_4_	26,760.7	62,238.0	2,817.87	7.2 × 10^−3^	10	0.14(3)	0.00106(12)	E2	1	5.04(3)	8
^3^F2_3_–^3^F1_4_	26,842.3	62,238.0	2,824.37	3.09 × 10^−1^	4	0.127(11)	0.0456(9)	M1 + E2	0.555(25)	−0.22(3)	4
^3^F2_4_–^3^F1_3_	26,973.7	62,364.3	2,824.78	7.14 × 10^−1^	3	0.267(11)	0.1039(12)	M1 + E2	0.801(12)	0.929(10)	3
^3^P2_2_–^3^P1_2_	26,468.2	61,854.1	2,825.15	5.00 × 10^−1^	2.4	0.338(5)	0.1411(24)	E2	0.99984(3)	−0.52(18)	2.4
^5^D_4_–^3^D_3_	1,282.7	36,630.0	2,828.24	4.1 × 10^−1^	9	−0.932(3)	0.5910(9)	M1	0.00234(5)	0.457(19)	9
^3^F2_4_–^3^F1_2_	26,973.7	62,321.1	2,828.23	1.63 × 10^−1^	7	0.503(8)	0.0220(12)	E2	1	0.29(4)	7
^5^D_4_–^1^G2_4_	1,282.7	36,585.6	2,831.80	4.8 × 10^−3^	18	−0.001868(12)	0.0049(5)	M1	0.000342(17)	0.250(11)	17
^3^F2_4_–^3^F1_4_	26,973.7	62,238.0	2,834.90	7.71 × 10^−1^	2.1	0.210(6)	0.114(3)	M1 + E2	0.971(5)	−0.02(16)	2.1
^3^F2_2_–^3^P1_2_	26,760.7	61,854.1	2,848.70	1.06 × 10^−2^	10	0.050(9)	0.0030(4)	M1 + E2	0.995(5)	4.45(4)	8
^3^F2_3_–^3^P1_2_	26,842.3	61,854.1	2,855.34	5.12 × 10^−2^	3	0.0505(25)	0.0145(6)	M1 + E2	0.982(4)	0.61(13)	3
^3^F2_4_–^3^P1_2_	26,973.7	61,854.1	2,866.10	2.44 × 10^−1^	2.5	0.0750(9)	0.0688(24)	E2	1	0.81(9)	2.5
^1^G2_4_–^1^G1_4_	36,585.6	71,280.3	2,881.44	1.14 × 10^−1^	7	0.081(7)	0.0241(16)	E2	0.9948(10)	0.38(6)	7
^1^I_6_–^1^G1_4_	37,511.6	71,280.3	2,960.46	2.99	1.9	−0.9848(9)	0.629(18)	E2	1	0.21(15)	1.9
^3^G_3_–^3^P1_1_	29,817.1	62,914.1	3,020.54	8.3 × 10^−3^	10	−0.0745(9)	0.00187(13)	E2	1	0.415(14)	10
^3^G_3_–^3^F1_3_	29,817.1	62,364.3	3,071.57	5.60 × 10 ^−1^	4	0.385(17)	0.0815(23)	M1 + E2	0.654(14)	0.55(3)	4
^3^G_3_–^3^F1_2_	29,817.1	62,321.1	3,075.65	1.130	2.0	0.33(3)	0.1525(7)	M1 + E2	0.724(23)	−0.67(16)	2.0
^3^G_3_–^3^F1_4_	29,817.1	62,238.0	3,083.53	5.5 × 10^−2^	8	0.277(19)	0.0081(5)	M1 + E2	0.564(20)	0.154(4)	8
^3^G_4_–^3^F1_3_	30,147.2	62,364.3	3,103.04	6.29 × 10^−1^	2.4	0.405(20)	0.0916(22)	M1 + E2	0.981(3)	−0.10(20)	2.4
^3^G_4_–^3^F1_2_	30,147.2	62,321.1	3,107.21	3.16 × 10^−1^	2.1	0.693(7)	0.0427(3)	E2	1	0.16(20)	2.1
^3^G_4_–^3^F1_4_	30,147.2	62,238.0	3,115.25	4.73 × 10^−1^	4	0.382(15)	0.0696(16)	M1 + E2	0.671(14)	0.651(20)	4
^3^P1_2_–^1^D1_2_	61,854.1	93,832.5	3,126.20	9.4 × 10^−2^	9	−0.00802(7)	0.00234(20)	M1	0.00695(21)	0.475(18)	9
^3^G_5_–^3^F1_3_	30,429.9	62,364.3	3,130.51	3.00 × 10^−1^	3	0.881(5)	0.0436(5)	E2	1	0.22(14)	3
^3^G_5_–^3^F1_4_	30,429.9	62,238.0	3,142.94	1.163	1.9	0.783(5)	0.1713(8)	M1 + E2	0.831(13)	0.38(17)	1.9
^3^F1_2_–^1^D1_2_	62,321.1	93,832.5	3,172.54	7.7 × 10^−2^	9	0.0451(10)	0.00191(16)	M1 + E2	0.0316(6)	0.451(19)	9
^3^F1_3_–^1^D1_2_	62,364.3	93,832.5	3,176.89	1.55 × 10^−1^	9	0.98985(9)	0.0038(3)	M1 + E2	0.0162(6)	0.447(20)	9
^5^D_2_–^3^G_4_	417.5	30,147.2	3,362.67	4.9 × 10^−5^	9	0.115(4)	0.000453(13)	E2	1	0.47(3)	9
^5^D_1_–^3^G_3_	142.4	29,817.1	3,368.91	3.3 × 10^−5^	10	0.153(8)	0.000225(6)	E2	1	0.41(3)	10
^5^D_3_–^3^G_5_	803.1	30,429.9	3,374.35	4.8 × 10^−5^	10	0.136(4)	0.000349(12)	E2	1	0.40(3)	10
^5^D_2_–^3^G_3_	417.5	29,817.1	3,400.43	8.4 × 10^−3^	18	0.2067(5)	0.057(4)	M1 + E2	0.0101(9)	0.252(10)	18
^5^D_3_–^3^G_4_	803.1	30,147.2	3,406.86	8.8 × 10^−3^	16	0.1012(4)	0.082(5)	M1 + E2	0.0207(15)	0.310(11)	16
^5^D_4_–^3^G_5_	1,282.7	30,429.9	3,429.88	8.2 × 10^−4^	15	0.782(12)	0.0060(4)	M1 + E2	0.326(21)	0.216(14)	15
^5^D_3_–^3^G_3_	803.1	29,817.1	3,445.62	2.0 × 10^−2^	18	0.01776(8)	0.136(11)	M1	0.00104(13)	0.254(10)	18
^5^D_4_–^3^G_4_	1,282.7	30,147.2	3,463.47	3.2 × 10^−2^	16	0.00535(3)	0.295(19)	M1	0.00051(8)	0.315(12)	16
^5^D_4_–^3^G_3_	1,282.7	29,817.1	3,503.54	2.9 × 10^−3^	18	−0.02342(14)	0.0197(16)	M1	0.000037(23)	0.251(10)	18
^3^H_4_–^1^F_3_	24,932.4	52,732.6	3,596.07	8.4 × 10^−3^	15	0.179(7)	0.0129(8)	M1 + E2	0.348(19)	0.214(10)	15
^3^H_5_–^1^F_3_	25,225.5	52,732.6	3,634.39	3.4 × 10^−3^	8	0.676(10)	0.00516(17)	E2	1	0.469(18)	8
^1^D1_2_–^1^S1_0_	93,832.5	121,130.1	3,662.28	5.07	1.9	0.9519(13)	0.0588(8)	E2	1	0.49(16)	1.9
^5^D_0_–^3^F2_2_	0.0	26,760.7	3,735.76	2.0 × 10^−5^	13	−0.0236(9)	0.000052(2)	E2	1	−0.62(3)	12 *
^5^D_1_–^3^F2_3_	142.4	26,842.3	3,744.27	4.7 × 10^−6^	7	−0.0040(3)	6.2(4) × 10^−6^	E2	1	0.33(5)	7
^3^D_2_–^3^P1_0_	36,758.2	63,419.8	3,749.65	1.058	1.9	0.761(7)	0.237(5)	E2	1	0.20(15)	1.9
^5^D_1_–^3^F2_2_	142.4	26,760.7	3,755.75	1.10 × 10^−1^	8	−0.955(3)	0.289(4)	M1	0.000223(7)	0.97(4)	8
^5^D_2_–^3^F2_4_	417.5	26,973.7	3,764.53	6 × 10^−8^	50	−0.00005(3)	6(4) × 10^−8^	E2	1	0.606(3)	50
^3^D_1_–^3^P1_0_	36,925.2	63,419.8	3,773.28	1.87 × 10^−1^	9	−0.1172(19)	0.042(3)	M1	0	0.462(18)	9
^5^D_0_–^3^P2_2_	0.0	26,468.2	3,777.05	7.6 × 10^−5^	8	0.0780(19)	0.000087(2)	E2	1	0.83(6)	8
^5^D_2_–^3^F2_3_	417.5	26,842.3	3,783.25	1.90 × 10^−1^	9	−0.24931(7)	0.251367(17)	M1	0.000088(4)	0.505(20)	9
^5^D_2_–^3^F2_2_	417.5	26,760.7	3,794.97	2.13 × 10^−1^	9	−0.1413(5)	0.561(4)	M1	1(3) × 10^−8^	0.463(20)	9
^5^D_1_–^3^P2_2_	142.4	26,468.2	3,797.48	4.1 × 10^−2^	8	−0.0174(3)	0.0466(10)	M1	0.00165(5)	−1.30(3)	8 *
^3^D_3_–^3^P1_1_	36,630.0	62,914.1	3,803.50	4.31 × 10^−1^	2.0	0.759(7)	0.097(3)	E2	1	0.27(15)	2.0
^3^P2_2_–^1^F_3_	26,468.2	52,732.6	3,806.35	1.21 × 10^−3^	19	0.34(10)	0.00185(18)	M1 + E2	0.0081(21)	0.156(10)	19
^5^D_3_–^3^F2_4_	803.1	26,973.7	3,820.00	1.80 × 10 ^−1^	9	−0.11327(12)	0.17361(9)	M1	0.000080(4)	0.510(20)	9
^3^D_2_–^3^P1_1_	36,758.2	62,914.1	3,822.14	1.044 × 10^−1^	2.3	0.778(9)	0.0236(3)	M1 + E2	0.985(3)	−0.10(20)	2.3
^5^D_2_–^3^P2_2_	417.5	26,468.2	3,837.58	4 × 10^−5^	200	0.00052(19)	0.00005(9)	M1 + E2	0.11(21)	0.096(2)	61 *
^5^D_3_–^3^F2_3_	803.1	26,842.3	3,839.27	4.9 × 10^−1^	9	−0.025879(5)	0.65408(6)	M1	4.55(15) × 10^−5^	0.504(20)	9
^3^D_1_–^3^P1_1_	36,925.2	62,914.1	3,846.71	5.41 × 10^−1^	3	0.457(18)	0.1224(12)	M1 + E2	0.676(19)	−0.04(5)	3
^3^F2_2_–^1^F_3_	26,760.7	52,732.6	3,849.22	1.87 × 10^−3^	9	0.297(6)	0.00287(6)	M1 + E2	0.940(8)	0.51(3)	9
^5^D_3_–^3^F2_2_	803.1	26,760.7	3,851.34	5.7 × 10^−2^	15	0.070(6)	0.150(8)	M1	0.000149(17)	−1.192(17)	13
^3^F2_3_–^1^F_3_	26,842.3	52,732.6	3,861.36	8.1 × 10^−3^	17	0.023(4)	0.0123(10)	M1 + E2	0.126(15)	0.218(12)	17
^1^G2_4_–^3^F1_3_	36,585.6	62,364.3	3,878.07	3.4 × 10^−2^	11	0.0160(4)	0.0050(5)	M1 + E2	0.059(3)	0.303(22)	11
^3^F2_4_–^1^F_3_	26,973.7	52,732.6	3,881.05	1.71 × 10^−2^	14	0.0254(14)	0.0263(13)	M1 + E2	0.138(9)	0.340(10)	14
^3^D_3_–^3^F1_3_	36,630.0	62,364.3	3,884.76	1.95 × 10 ^−1^	7	0.0180(11)	0.0284(18)	M1 + E2	0.072(5)	0.602(21)	7
^5^D_4_–^3^F2_4_	1,282.7	26,973.7	3,891.31	8.5 × 10^−1^	9	−0.008406(8)	0.8191(4)	M1	0.000170(4)	0.509(20)	9
^3^D_3_–^3^F1_2_	36,630.0	62,321.1	3,891.30	4.8 × 10^−2^	9	0.0497(6)	0.0065(5)	M1 + E2	0.079(5)	0.386(15)	9
^5^D_3_–^3^P2_2_	803.1	26,468.2	3,895.24	7.9 × 10^−1^	8	−0.957(18)	0.901(4)	M1	0.000177(5)	0.85(3)	8
^1^G2_4_–^3^F1_4_	36,585.6	62,238.0	3,897.17	3.8 × 10^−2^	10	0.01470(24)	0.0056(5)	M1 + E2	0.0778(22)	0.327(25)	10
^3^D_3_–^3^F1_4_	36,630.0	62,238.0	3,903.92	2.61 × 10^−1^	8	0.910(12)	0.038(3)	M1 + E2	0.102(9)	0.391(18)	8
^3^D_2_–^3^F1_3_	36,758.2	62,364.3	3,904.21	1.35 × 10^−2^	3	0.083(4)	0.00197(6)	E2	0.99990(8)	0.79(15)	3
^3^D_2_–^3^F1_2_	36,758.2	62,321.1	3,910.81	3.08 × 10^−1^	8	0.0663(12)	0.042(3)	M1 + E2	0.052(4)	0.479(18)	8
^5^D_4_–^3^F2_3_	1,282.7	26,842.3	3,911.32	7.0 × 10^−2^	8	0.03872(4)	0.09226(13)	M1	0.000442(10)	0.519(20)	8
^5^D_4_–^3^F2_2_	1,282.7	26,760.7	3,923.84	1.53 × 10^−6^	11	0.0242(11)	4.03(16) × 10^−6^	E2	1	−1.38(4)	10 *
^3^D_1_–^3^F1_2_	36,925.2	62,321.1	3,936.53	2.07 × 10^−1^	8	0.895(8)	0.0279(19)	M1 + E2	0.094(8)	0.402(18)	8
^3^D_3_–^3^P1_2_	36,630.0	61,854.1	3,963.34	5.91 × 10^−1^	2.3	0.870(5)	0.1668(16)	M1 + E2	0.793(13)	0.20(13)	2.3
^5^D_4_–^3^P2_2_	1,282.7	26,468.2	3,969.42	9.7 × 10^−6^	9	−0.0060(3)	1.11(5) × 10^−5^	E2	1	0.56(3)	9
^3^D_2_–^3^P1_2_	36,758.2	61,854.1	3,983.59	3.56 × 10^−1^	4	0.376(23)	0.1006(8)	M1 + E2	0.641(23)	−0.39(5)	4
^1^D2_2_–^1^G1_4_	46,291.1	71,280.3	4,000.60	1.36 × 10^−2^	5	0.0258(14)	0.00286(16)	E2	1	0.34(5)	5
^5^D_0_–^3^P2_1_	0.0	24,972.8	4,003.22	1.37 × 10^−1^	8	−0.0323(3)	0.1033(10)	M1	0	0.612(22)	8
^3^D_1_–^3^P1_2_	36,925.2	61,854.1	4,010.27	7.56 × 10^−2^	2.5	0.379(20)	0.02134(18)	M1 + E2	0.709(18)	0.13(12)	2.5
^5^D_1_–^3^P2_1_	142.4	24,972.8	4,026.18	2.7 × 10^−4^	12	0.073(3)	0.000201(7)	M1 + E2	0.625(19)	0.275(13)	12
^5^D_2_–^3^P2_1_	417.5	24,972.8	4,071.29	1.18	9	−0.2726(10)	0.8860(19)	M1	0.000131(3)	0.454(19)	9
^5^D_2_–^3^H_4_	417.5	24,932.4	4,078.00	8.0 × 10^−8^	7	0.0014(3)	0.000014(5)	E2	1	2.59(4)	7
^5^D_3_–^3^H_5_	803.1	25,225.5	4,093.45	1.52 × 10^−6^	6	0.0106(5)	0.00225(12)	E2	1	1.00(7)	6
^5^D_4_–^3^H_6_	1,282.7	25,528.4	4,123.28	1.16 × 10^−5^	10	0.0308(4)	0.0185(18)	E2	1	0.40(3)	10
^5^D_3_–^3^P2_1_	803.1	24,972.8	4,136.25	5.5 × 10^−5^	9	0.03538(23)	4.11(8) × 10^−5^	E2	1	0.44(3)	9
^5^D_3_–^3^H_4_	803.1	24,932.4	4,143.17	9.2 × 10^−4^	25	0.1288(6)	0.1629(3)	M1	0.000017(4)	0.170(9)	24
^5^D_4_–^3^H_5_	1,282.7	25,225.5	4,175.44	1.2 × 10^−5^	27	0.9716(20)	0.018(5)	M1 + E2	0.0285(19)	0.167(7)	26
^5^D_1_–^3^P2_0_	142.4	24,055.5	4,180.63	1.52	9	−0.1224(6)	0.999728(5)	M1	0	0.407(18)	9
^5^D_4_–^3^H_4_	1,282.7	24,932.4	4,227.19	4.7 × 10^−3^	25	0.01030(7)	0.8368(3)	M1	0.000280(7)	0.170(9)	24
^5^D_2_–^3^P2_0_	417.5	24,055.5	4,229.29	4.1 × 10^−4^	9	0.1029(5)	0.000272(5)	E2	1	0.41(3)	9
^1^S2_0_–^3^P1_1_	39,633.0	62,914.1	4,294.12	1.57 × 10^−1^	8	−0.0827(14)	0.0355(22)	M1	0	0.50(3)	8
^3^G_3_–^1^F_3_	29,817.1	52,732.6	4,362.63	1.40 × 10^−1^	9	−0.01243(24)	0.2141(7)	M1	0.00700(18)	0.484(20)	9
^3^G_4_–^1^F_3_	30,147.2	52,732.6	4,426.40	1.93 × 10^−1^	8	0.436(11)	0.2955(23)	M1	0.00030(6)	0.540(21)	8
^1^G1_4_–^1^D1_2_	71,280.3	93,832.5	4,432.91	5.54 × 10^−1^	2.0	0.867(5)	0.01377(11)	E2	1	0.46(16)	2.0
^3^G_5_–^1^F_3_	30,429.9	52,732.6	4,482.50	9.1 × 10^−4^	10	0.813(6)	0.00139(3)	E2	1	0.343(17)	10
^3^P2_1_–^1^D2_2_	24,972.8	46,291.1	4,689.49	7.6 × 10^−2^	8	0.313(5)	0.05932(17)	M1	0.00191(5)	0.494(19)	9
^3^P2_2_–^1^D2_2_	26,468.2	46,291.1	5,043.26	2.14 × 10^−1^	8	0.0215(6)	0.1662(22)	M1	0.000072(3)	1.33(5)	8
^3^F2_2_–^1^D2_2_	26,760.7	46,291.1	5,118.80	2.48 × 10^−1^	9	−0.03653(17)	0.193(3)	M1	0.000119(4)	−0.316(18)	9 *
^3^F2_3_–^1^D2_2_	26,842.3	46,291.1	5,140.27	4.9 × 10^−1^	8	0.999725(23)	0.3795(19)	M1	0.00106(3)	0.578(22)	8
^1^F_3_–^1^G1_4_	52,732.6	71,280.3	5,390.01	1.108 × 10^−1^	1.9	0.920(3)	0.0233(6)	M1 + E2	0.981(3)	0.25(14)	1.9
^1^D2_2_–^3^P1_1_	46,291.1	62,914.1	6,014.10	1.33 × 10^−1^	9	0.99741(21)	0.0301(20)	M1	0.00256(6)	0.408(25)	9
^3^G_3_–^1^D2_2_	29,817.1	46,291.1	6,068.49	1.26 × 10^−2^	17	−0.99957(4)	0.0098(9)	M1	0.0031(4)	0.269(11)	17
^1^G2_4_–^1^F_3_	36,585.6	52,732.6	6,191.4	1.176 × 10^−2^	2.1	0.2411(13)	0.0180(17)	E2	0.9942(13)	0.31(17)	2.1
^3^D_3_–^1^F_3_	36,630.0	52,732.6	6,208.46	1.82 × 10^−1^	9	0.019892(22)	0.2794(12)	M1	0.000269(7)	0.431(18)	9
^1^D2_2_–^3^F1_3_	46,291.1	62,364.3	6,219.82	7.4 × 10^−2^	9	−0.253(3)	0.0107(9)	M1	0.00210(5)	0.371(15)	9
^1^D2_2_–^3^F1_2_	46,291.1	62,321.1	6,236.58	5.6 × 10^−2^	10	0.02148(14)	0.0076(6)	M1	0.000023(3)	0.356(16)	10
^3^D_2_–^1^F_3_	36,758.2	52,732.6	6,258.28	8.2 × 10^−2^	9	0.815(6)	0.1251(4)	M1	0.00223(5)	0.462(19)	9
^1^D2_2_–^3^P1_2_	46,291.1	61,854.1	6,423.72	1.88 × 10^−1^	9	0.03917(5)	0.053(3)	M1	0.000694(21)	0.421(25)	9
^3^P2_1_–^1^S2_0_	24,972.8	39,633.0	6,819.3	1.66	8	−0.999980(2)	0.99912(6)	M1	0	0.615(22)	8
^3^P2_0_–^3^D_1_	24,055.5	36,925.2	7,768.1	5.7 × 10^−2^	9	0.9999895(11)	0.0778(6)	M1	0	0.50(3)	9
^3^H_5_–^1^I_6_	25,225.5	37,511.6	8,137.0	1.24 × 10^−1^	9	1.0000000(0)	0.4314(3)	M1	1.4(5) × 10^−7^	0.492(20)	9
^3^H_6_–^1^I_6_	25,528.4	37,511.6	8,342.7	1.63 × 10^−1^	9	0.0059430(0)	0.56728(17)	M1	1.14(4) × 10^−5^	0.479(20)	9
^3^P2_1_–^3^D_1_	24,972.8	36,925.2	8,364.2	1.34 × 10^−1^	8	0.2511591(3)	0.1833(14)	M1	0.0041(4)	0.50(3)	8
^3^P2_1_–^3^D_2_	24,972.8	36,758.2	8,482.7	7.2 × 10^−4^	16	0.0076(9)	0.00125(9)	M1 + E2	0.098(16)	0.155(16)	16
^3^H_4_–^1^G2_4_	24,932.4	36,585.6	8,579.0	1.74 × 10^−1^	7	0.0082(3)	0.177(3)	M1	4.87(13) × 10^−5^	1.02(3)	7
^3^H_5_–^1^G2_4_	25,225.5	36,585.6	8,800.3	2.54 × 10^−1^	8	−0.741(12)	0.2584(22)	M1	0.000105(3)	0.68(3)	8
^3^P2_2_–^3^D_1_	26,468.2	36,925.2	9,560.3	3.7 × 10^−2^	9	−0.1111(10)	0.0513(8)	M1	0.0031(5)	0.88(3)	9
^3^P2_2_–^3^D_2_	26,468.2	36,758.2	9,715.5	5.9 × 10^−2^	10	0.01476(11)	0.1024(17)	M1	0.0033(3)	−0.058(23)	10
^3^F2_2_–^3^D_1_	26,760.7	36,925.2	9,835.5	1.80 × 10^−2^	11	0.88(3)	0.0246(6)	M1 + E2	0.111(12)	−0.63(3)	10 *
^3^P2_2_–^3^D_3_	26,468.2	36,630.0	9,838.1	6.1 × 10^−2^	9	−0.945(6)	0.0869(12)	M1	0.0037(4)	0.42(3)	9
^3^F2_2_–^3^D_2_	26,760.7	36,758.2	9,999.8	1.75 × 10^−2^	8	0.067(6)	0.0303(7)	M1 + E2	0.041(4)	1.23(6)	8
^3^F2_3_–^3^D_2_	26,842.3	36,758.2	10,082.0	2.96 × 10^−3^	8	0.36(3)	0.00511(9)	M1+E2	0.55(4)	−0.404(23)	8
^3^F2_3_–^3^D_3_	26,842.3	36,630.0	10,214.1	7.5 × 10^−3^	8	0.048(6)	0.01083(10)	M1 + E2	0.069(6)	0.370(18)	8
^3^F2_3_–^1^G2_4_	26,842.3	36,585.6	10,260.7	1.46 × 10^−1^	7	0.599(18)	0.1487(21)	M1	1.50(8) × 10^−6^	0.91(3)	7
^3^D_3_–^1^D2_2_	36,630.0	46,291.1	10,348.0	1.16×10^−1^	9	0.9537(17)	0.0902(8)	M1	0.000194(5)	0.473(21)	9
^3^F2_4_–^3^D_3_	26,973.7	36,630.0	10,353.1	9.7 × 10^−3^	6	0.399(11)	0.0139(4)	M1 + E2	0.214(14)	0.451(20)	6
^1^F_3_–^3^F1_3_	52,732.6	62,364.3	10,379.5	1.24 × 10^−2^	9	0.001661(3)	0.00181(14)	M1	0.000142(7)	0.430(18)	9
^3^F2_4_–^1^G2_4_	26,973.7	36,585.6	10,400.9	3.3 × 10^−1^	9	0.01357(12)	0.336(3)	M1	3.6(7) × 10^−7^	0.453(20)	9
^1^F_3_–^3^F1_2_	52,732.6	62,321.1	10,426.3	1.88 × 10^−1^	8	0.9999919(9)	0.0254(18)	M1	1.35(4) × 10^−5^	0.441(19)	8
^3^D_2_–^1^D2_2_	36,758.2	46,291.1	10,487.1	2.17 × 10^−2^	9	0.006205(20)	0.01686(16)	M1	0.000181(6)	0.432(20)	9
^1^F_3_–^3^F1_4_	52,732.6	62,238.0	10,517.5	1.02 × 10^−1^	8	0.999963(4)	0.0151(11)	M1	0.000285(8)	0.445(19)	8
^3^D_1_–^1^D2_2_	36,925.2	46,291.1	10,674.1	1.04 × 10^−1^	9	0.9869(6)	0.0804(7)	M1	2.24(8) × 10^−5^	0.471(21)	9
^3^F1_4_–^1^G1_4_	62,238.0	71,280.3	11,056.1	5.7 × 10^−2^	9	−0.012325(3)	0.0120(7)	M1	3.74(8) × 10^−5^	0.491(22)	9
^3^F1_3_–^1^G1_4_	62,364.3	71,280.3	11,212.7	3.3 × 10^−2^	9	−1.0000000(0)	0.0069(4)	M1	0.00102(3)	0.492(22)	9
^3^G_3_–^3^D_1_	29,817.1	36,925.2	14,064.6	6.68 × 10^−4^	2.1	0.753(9)	0.00091(8)	E2	1	−0.05(19)	2.1
^3^G_5_–^1^I_6_	30,429.9	37,511.6	14,117.0	3.6 × 10^−4^	17	1.0000000(0)	0.00126(10)	M1	0.0032(3)	0.264(11)	17
^3^G_3_–^1^G2_4_	29,817.1	36,585.6	14,770.3	4.2 × 10^−2^	12 *	−1.0000000(0)	0.0424(18)	M1	2.26(10) × 10^−5^	0.305(14)	12
^3^G_4_–^1^G2_4_	30,147.2	36,585.6	15,527.6	5.9 × 10^−3^	14	−0.00145(4)	0.0060(4)	M1	0.000037(4)	0.322(13)	14
^3^G_5_–^1^G2_4_	30,429.9	36,585.6	16,240.7	2.5 × 10^−2^	12 *	−1.0000000(0)	0.0255(9)	M1	1.21(4) × 10^−5^	0.333(17)	12
^3^H_4_–^3^G_5_	24,932.4	30,429.9	18,185.1	1.00 × 10^−3^	2.5	0.0044(3)	0.0073(5)	M1	0.000031(11)	1.32(18)	2.5
^3^H_4_–^3^G_4_	24,932.4	30,147.2	19,171.0	3.44 × 10^−2^	4	−0.0093(5)	0.321(19)	M1	0.000297(17)	3.40(8)	4
^3^H_5_–^3^G_5_	25,225.5	30,429.9	19,209.3	4.7 × 10^−2^	8	−0.0055920(0)	0.344(3)	M1	0.000144(16)	0.57(3)	8
^3^H_5_–^3^G_4_	25,225.5	30,147.2	20,318	6.6 × 10^−4^	19 *	0.080(22)	0.0061(6)	M1 + E2	0.102(21)	0.156(11)	19
^3^H_6_–^3^G_5_	25,528.4	30,429.9	20,402	4.7 × 10^−2^	8	1.0000000(0)	0.344(3)	M1	0.00156(13)	0.55(3)	8
^3^H_4_–^3^G_3_	24,932.4	29,817.1	20,472	4.2 × 10^−2^	6	−0.642(22)	0.283(12)	M1	0.00167(12)	0.97(4)	6
^3^F2_4_–^3^G_5_	26,973.7	30,429.9	28,934	4.0 × 10^−2^	8	1.0000000(0)	0.295(5)	M1	0.000053(5)	0.72(3)	8
^3^F2_3_–^3^G_4_	26,842.3	30,147.2	30,258	9.7 × 10^−4^	19 *	0.0071(8)	0.0090(9)	M1	0.0013(3)	0.267(10)	19
^3^F2_4_–^3^G_4_	26,973.7	30,147.2	31,511	2.99 × 10^−2^	7	−0.00800(5)	0.278(6)	M1	3.7(6) × 10^−6^	0.84(3)	7
^3^F2_2_–^3^G_3_	26,760.7	29,817.1	32,718	3.3 × 10^−2^	9	−1.0000000(0)	0.225(3)	M1	0.000029(3)	0.572(24)	9
^3^F2_3_–^3^G_3_	26,842.3	29,817.1	33,616	4.1 × 10^−2^	9	−0.0332325(9)	0.278(4)	M1	4.4(5) × 10^−6^	0.591(24)	9
^3^F2_4_–^3^G_3_	26,973.7	29,817.1	35,169	1.6 × 10^−4^	22 *	0.0015(4)	0.0011(4)	M1	0.0008(4)	1.015(7)	22
^3^H_4_–^3^F2_4_	24,932.4	26,973.7	48,988	6.3 × 10^−3^	15 *	−0.03761(23)	0.0061(4)	M1	1.7(10) × 10^−8^	0.286(19)	15
^3^H_4_–^3^F2_3_	24,932.4	26,842.3	52,359	1.6 × 10^−3^	15 *	−1.0000000(0)	0.00218(17)	M1	9.1(6) × 10^−6^	0.286(17)	15
^3^P2_1_–^3^P2_2_	24,972.8	26,468.2	66,872	4.5406 × 10^−2^	0.06	1.0000000(0)	0.052(4)	M1	9.9(6) × 10^−8^	2621(13)	0.013
^3^P1_2_–^3^P1_1_	61,854.1	62,914.1	94,340	2.6842 × 10^−2^	0.012	1.0000000(0)	0.00607(17)	M1	6.95(15) × 10^−7^	444(16)	0.012
^3^P2_0_–^3^P2_1_	24,055.5	24,972.8	109,020	1.3837 × 10^−2^	0.06	1.0000000(0)	0.0104(9)	M1	0	35(3)	0.06
^3^P1_1_–^3^P1_0_	62,914.1	63,419.8	197,700	7.0044 × 10^−3^	0.010	1.0000000(0)	0.00157(4)	M1	0	453(15)	0.010
^5^D_3_–^5^D_4_	803.1	1,282.7	208,500	2.9885 × 10^−3^	0.007	1.0000000(0)	0.9999994364(2)	M1	1.048(20) × 10^−7^	594(20)	0.007
^5^D_2_–^5^D_3_	417.5	803.1	259,300	2.6639 × 10^−3^	0.010	1.0000000(0)	0.99999968(8)	M1	3.61(7) × 10^−8^	325(13)	0.011
^3^G_3_–^3^G_4_	29,817.1	30,147.2	302,900	9.212 × 10^−4^	0.23	1.0000000(0)	0.0086(8)	M1	3.1(6) × 10^−10^	17.5(6)	0.23
^3^H_5_–^3^H_6_	25,225.5	25,528.4	330,100	6.144 × 10^−4^	0.15	1.0000000(0)	0.9815(18)	M1	8(3) × 10^−11^	24.6(6)	0.15
^3^H_4_–^3^H_5_	24,932.4	25,225.5	341,200	6.625 × 10^−4^	0.15	1.0000000(0)	0.979(5)	M1	2.8(4) × 10^−10^	49(3)	0.15
^3^G_4_–^3^G_5_	30,147.2	30,429.9	353,700	4.685 × 10^−4^	0.4	1.0000000(0)	0.0034(3)	M1	7.4(11) × 10^−10^	7.7(4)	0.4
^5^D_1_–^5^D_2_	142.4	417.5	363,500	1.1839 × 10^−3^	0.015	1.0000000(0)	0.99999992(8)	M1	6.89(13) × 10^−9^	294(11)	0.015
^5^D_0_–^5^D_1_	0.0	142.4	702,000	1.5517 × 10^−4^	0.018	1.0000000(0)	1.000000000(0)	M1	0	264(7)	0.018

a The energy levels and Ritz wavelengths calculated from them are taken from [5]. Wavelengths between 2,000 Å and 20,000 Å are in standard air; shorter and longer wavelengths are in vacuum;

b Relative standard deviation of straight *A* values over 10,000 trial calculations (percent). Transitions for which the accuracy estimated in [5] should be degraded are marked by an asterisk in this column;

c Cancellation factors for mixed M1 + E2 transitions are calculated as a weighted mean of absolute values, CF^M1+E2^ = (|CF^M1^|*A*^M1^ + |CF^E2^|*A*^E2^)/(*A*^M1^ + *A*^E2^). The quantity in parentheses is the standard deviation of CF over 10,000 trial calculations (in the units of the last digit of the value);

d Branching fractions were calculated for each trial. The given value is the result of the initial LSF calculation. The quantity in parentheses is the standard deviation of the branching fraction over 10,000 trial calculations (in the units of the last digit of the value);

e Transition type is specified as mixed M1 + E2 for transitions having the fraction of the minor contribution to the total *A* value greater than 1%;

f Fraction of E2 transition in the total *A* value was calculated for each trial. The given value is the result of the initial LSF calculation. The quantity in parentheses is the standard deviation of the E2 fraction over 10,000 trial calculations (in the units of the last digit of the value);

g The parameter *p* of the optimal Box–Cox transformation is determined as a weighted mean over five runs, four with 1,000 trials each and one with 10,000 trials. The quantity in parentheses is the weighted standard deviation of the mean over five runs;

i Relative standard deviation of transformed *A* values using the optimal Box–Cox transformation with the given parameter *p*, over 10,000 trial calculations (percent). The starred values denote transitions for which the optimal Box–Cox transformation yields statistical distributions that are far from normal.
